# Residency and space use estimation methods based on passive acoustic telemetry data

**DOI:** 10.1186/s40462-022-00364-z

**Published:** 2023-03-01

**Authors:** S. Kraft, M. Gandra, R. J. Lennox, J. Mourier, A. C. Winkler, D. Abecasis

**Affiliations:** 1 Center of Marine Sciences (CCMAR), Universidade do Algarve, Faro, Portugal; 2 Laboratory for Freshwater Ecology and Inland Fisheries at NORCE Norwegian Research Center, Bergen, Norway; 3 Norwegian Institute for Nature Research (NINA), Trondheim, Norway; 4 MARBEC, Univ Montpellier, CNRS, Ifremer, IRD, Sète, France; 5 Department of Ichthyology and Fisheries Science, Rhodes University, Makhanda, South Africa

**Keywords:** Home range, Range distribution, Movement ecology, Biotelemetry, Data analysis

## Abstract

Acoustic telemetry has helped overcome many of the challenges faced when studying the movement ecology of aquatic species, allowing to obtain unprecedented amounts of data. This has made it into one of the most widely used methods nowadays. Many ways to analyse acoustic telemetry data have been made available and deciding on how to analyse the data requires considering the type of research objectives, relevant properties of the data (e.g., resolution, study design, equipment), habits of the study species, researcher experience, among others. To ease this decision process, here we showcase (1) some of the methods used to estimate pseudo-positions and positions from raw acoustic telemetry data, (2) methods to estimate residency and (3) methods to estimate two-dimensional home and occurrence range using geometric or hull-based methods and density-distribution methods, a network-based approach, and three-dimensional methods. We provide examples of some of these were tested using a sample of real data. With this we intend to provide the necessary background for the selection of the method(s) that better fit specific research objectives when using acoustic telemetry.

**Supplementary Information:**

The online version contains supplementary material available at 10.1186/s40462-022-00364-z.

## Introduction

Movement is a central and complex component of animal life [141]. Many metrics have been developed to quantify movement. Two of the most common ones are residency and space use measurements, like core and home range areas. Residency can be defined as an individual’s preference for an area where it decides to stay over a specified and usually extended period, which is mostly occupied uninterruptedly [37]. Generally, brief departures from this area can occur and are considered part of resident behaviour. Animals can also display site fidelity if despite being absent for a long time they return to the same area, which is different from the brief forays to other places mentioned earlier [37]. The duration of this absence is not a fixed value and is allowed to vary, but is generally expected to be similar or longer than the residency in said area [37]. How we define residency can therefore be also adapted to a specific time frame defined by the analyst”. The concept of residency, which prompts familiarity with the distribution of resources in a defined area, is tightly related to home range [161].

Although there is no universal definition for the concept of home range [163], one of the most frequently cited ones is that of Burt [26]:

“that area traversed by the individual in its normal activities of food gathering, mating, and caring for young. Occasional sallies outside the area, perhaps exploratory in nature, should not be considered as in part of the home range.” […] “The size of the home range may vary with sex, possibly age, and season.”

Home range estimation is an attempt at quantifying an animal’s relationship with its environment and is a challenging task [163]. This is commonly done by modelling space use to evaluate how an animal occupies space and is influenced by environmental and biotic factors [117, 164, 169]. Conventionally, two levels (or isopleths) of space use are reported, the core- or 50% area [64], which refers to the most frequently visited part of a range containing the features most important to the individual, and the home range or 95% area [108], which fits the traditional definition of home range. However, despite vast technological improvements in acoustic telemetry, no existing technique allows recording continuous long-term data without gaps or errors, and home range should be studied using methods that include these sources of uncertainty [117]. Examples of this are location error and the variation in detection range of acoustic receivers (hereon “receivers”), gaps from irregular or spaced out sampling, and missing data resulting from technical errors like code collisions, loss of receivers, battery failure, among others [115, 132, 154]. Home range estimation methods need to be objective, repeatable, and make biological sense [163]. Disagreement about what measurements satisfy these criteria has led to the development of several methods over the years, and new ones are constantly emerging. The approach of most home range estimators can be classified into two general categories: they can be either geometric, which is based on constructing hulls to outline the animal’s home range and lack a probabilistic basis, or statistical, some of which use utilization distributions, which describes the intensity of use given to different areas by an animal [76].

The study of movement ecology in aquatic environments has been historically challenging, however, acoustic telemetry has helped overcome this and is one of the most widely used methods nowadays [103]. A passive acoustic telemetry setup is commonly composed of three main elements: receivers, acoustic transmitters (hereon, transmitters or tags), and individuals we wish to study. Each individual is fitted with a transmitter that emits a uniquely coded signal. When a tagged animal comes into the detection range of a receiver, the emitted signal is picked up and stored, along with the time and date of detection and any additional information the specific tag model might collect, such as pressure, temperature or acceleration. To collect this information receivers are deployed at known locations to create a detection array throughout an area of monitoring interest. Receivers in these configurations usually have overlapping detection ranges for position estimation purposes, to monitor all movements in an area, or to act as gates to detect of crossing movements [99]. Some systems can be adequately covered by a comprehensive network of receivers with overlapping detection ranges, which allow triangulation or multilateralization of the acoustic signals to calculate a position and derive a path, which is a special case of acoustic telemetry providing precise positions. The main advantages of acoustic telemetry are the ability to constantly and simultaneously monitor many active tags, and its cost-effectiveness considering a running array’s low maintenance effort and the amount of data it can provide [99]. Like any methodology, it also has some limitations that should be accounted for. Detections cannot be recorded in areas out of the detection range of the receivers, which can bias the estimated area use of the tagged animal if it is present in areas where it cannot be detected. The fate of undetected animals with active tags is in most cases undetermined, as it can be attributed to different reasons, e.g., Klinard and Matley [118]. Additionally, while the successful detection of a tag on a receiver confirms its presence within detection range, the distance from the receiver, and the position of the animal, are unobserved. Finally, the detection ranges of receivers fluctuate unpredictably in four dimensions with environmental noise, water stratification resulting from temperature and salinity gradients, and other environmental factors [115, 154].

This review showcases some of the estimators of position, residency, and space use with a focus on their application to data derived from passive acoustic telemetry systems. This way, we hope to provide background to guide a more thorough selection of the method(s) that better fit specific research objectives in acoustic telemetry. Some of the described methods were tested using a subsample of tracking data from thornback rays, *Raja clavata*, (abacus plots), and a common stingray, *Dasyatis pastinaca*, for the space use estimators. These data sets were used to illustrate the output of some of the methods and in some instances to show differences between them. However, it must be noted that since real life data is being used, not all differences described for each method might be noticeable in the figures.

## Methods

The search for literature where residency, position, and home range estimation methods were described and/or applied was performed using Google Scholar. The methods for each of these three categories (position, residency, and space use estimation) were looked for with a set of keywords that were used in varying combinations. Some keywords were used in all searches (either “acoustic telemetry”, “acoustic tracking”, or “acoustic monitoring” with “estimation”) which were used in combination with words exclusive to each of the three search categories. The keywords exclusive to residency were “residence”, “residency”, “index”, and “site fidelity”; those exclusive to position estimation were “positioning” and “fine-scale”; while the exclusive words for the home range search were “home range”, “contour area”, “core area”, and “home range area”. Additional literature was also drawn from the citations in the manuscripts found using Google Scholar.

### Abacus plots

**Fig. 1:**
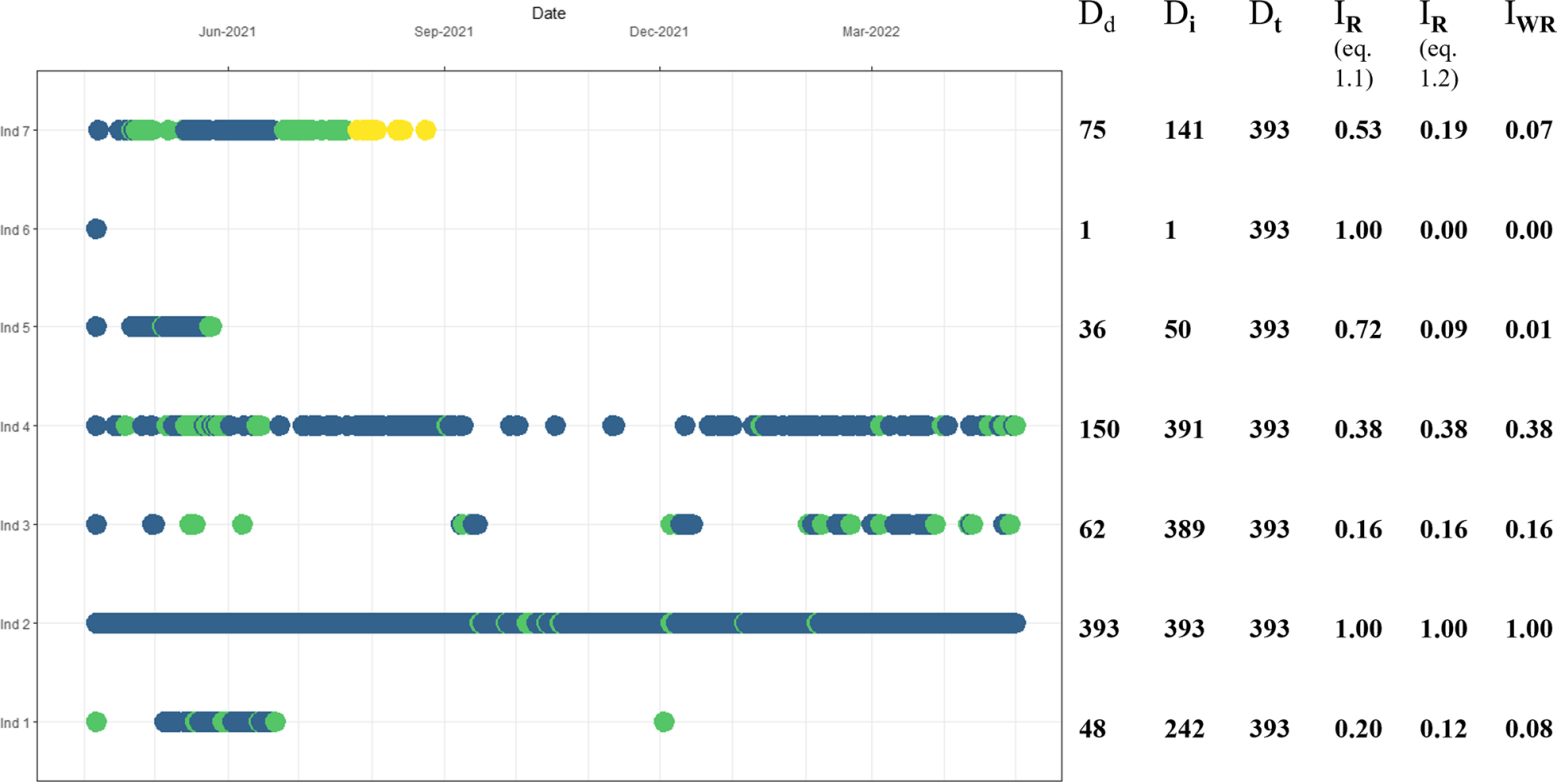
Abacus plot showing the detection patterns of seven Raja clavata individuals, from 06/04/2021 to 03/05/2022. Their respective days detected (D_d_), detection interval (D_i_), monitoring/study period (D_t_), and residency indices calculated with the three described fractions are also shown: I_R_ as Eq. 1 (D_d_/D_i_).; I_R_ as Eq. 2 (D_d_/D_t_); and I_WR_ = (D_d_/D_t_) × (D_i_/D_t_). The colours illustrate how different arrays or different sections in the same array can be colour-coded to add extra information to the plot

Abacus plots or calendar plots are an informative and simple way to undertake initial explorations of acoustic telemetry data and obtain a general idea of the residency of each animal, and even dispersion in some cases. The frequency and spread of detections can be visualized over time like a chronogram, which can provide an idea of how many times each individual was detected, their permanence in the study area and coarse movement patterns among receivers or areas in the array. In an abacus plot, time is displayed on the X-axis and the Y-axis represents either receivers or tagged individuals on individual lines, over which detections are represented as dots (Fig. 1). However, this provides no information about the spatial configuration of the array. For linear systems such as rivers, longitude or latitude may be replaced by receiver number to generate a spatial abacus plot. Otherwise, a best practice would be to sort receivers by some spatial metric such as distance from a point of interest. Single-individual plots feature the receivers at which each animal was detected, showing the movement across receivers, while plots with more than one animal separately present each animal’s cumulative detections. This latter format trades detailed information of movement between receivers for a more general view by condensing all detections into a single figure. More information can be added in many ways to facilitate the identification of patterns. Detections can be color-coded to include spatial or temporal data, dot size can be set to represent the number of detections in a day, or when receivers are displayed on the Y-axis dot size can represent the number of different tags detected within a defined time window. Spatial information can be included by coding detections according to receivers or sub-arrays of a particular subsection of the full array at which the animal was detected. Colour coding can follow seasons or day/night regimes and will ultimately depend on the time range of the data. Finally, both spatial and temporal information can be combined for example by colour coding detections by area and shading the background with different colours to reflect seasons of the year or time of day. These plots can also be used to identify unusual detection patterns, for example in the event of capture by fishing or post-surgical mortality or tag loss [118, 189], even in the absence of predation or accelerometer tags to support it [88, 201].

#### Residency estimation

##### Residency index

The residency index (I_R_) has two forms. The total number of days the animal was detected (D_d_) can be divided by either (1) detection interval, the number of days between first and last detection (D_i_) or (2) monitoring or study interval, the total number of monitoring days in the study (time between tagging date and last monitoring day) (D_t_) (Eq. 1). The resulting value fluctuates from 0 (no residency) to 1 (full residency) [2]. Residency can be calculated at any spatial or temporal scale, as D_d_ can correspond from the entire array to individual receivers. Similarly, data can be partitioned into timeframes to calculate seasonal or monthly residency. This residency index can be adapted to represent other time frames, like the number of hours in which at least one detection was made per day.

1.1 $$I_{R} = \frac{{D_{d} }}{{D_{i} }}$$

1.2 $$I_{R} = \frac{{D_{d} }}{{D_{t} }}$$

Both forms of the residency index can be interpreted differently and present some considerations [39]. In Eq. 1.1, using D_i_ accounts for tag loss, which ensures the calculation includes the period for which one knows the animal was alive and the tag operational. It represents a maximum residency value [39]. The approach of Eq. 1.2 is more conservative and gives a minimum residency value. The study period duration D_t_ is used to estimate the index and assumes that throughout it the animal was alive and detectable when in range.

However, Eq. 1.1 can in some cases overestimate residency. When a short detection interval is obtained (first and last detection days are close to each other), a high residency index for that period is obtained. Unless it was the objective, this approach can be troublesome when the study period (D_t_) is much longer. Similar residency values can be obtained for two individuals detected with a similar consistency in the study area but over two very different time intervals [39]. For example, similar values can be obtained by an individual detected 4 days over a period of 5 days and an individual detected 48 days over 60 days, however, the latter was present for a much longer time when considering the duration of the study period.

On the other hand, residency values estimated using Eq. 2 can be biased upwards for animals that were tagged later during the study [39]. Similarly, the absence of detections is assumed to be because the animal is out of detection range, without considering alternative scenarios that can lead to a cessation in detections, like death from predation or fishing.

The occurrence of events that lead to changes in detection pattern, like fishing or predation, can be assessed by observing individual detection plots [88, 118, 189, 201]. For example, an individual would suddenly cease transmitting after being fished, while suffering predation can produce a change in the detection pattern to reflect the movement of the predator. On the other hand, a different pattern is produced after events that result in the animal/tag to remain static on the bottom, e.g., natural mortality, partial/failed predation, fishing discard, tag loss. In this case, only the receiver(s) covering that area can detect the tag, so it stops being detected elsewhere. A static tag’s detection frequency can also increase on account of being permanently within detection range. If these situations are unaccounted for, the real residency values can be artificially modified (e.g., increased by a static tag on the bottom).

##### Weighted residency index

The weighted residency index (I_WR_) is composed of two fractions (Eq. 2) and ranges from 0 to 1. The first fraction corresponds to D_d_ divided by D_t_, which is weighted by a second fraction, the period between first and last detections (D_i_) divided by D_t_ [124]. This formula can also represent residency at various spatial levels by adjusting D_d_ to represent the detections obtained at the chosen scale.

2 $$I_{WR} = \frac{{D_{d} }}{{D_{t} }} \times \frac{{D_{i} }}{{D_{t} }}$$

If the tag lifetime is shorter than the total monitoring period, the value of D_t_ should be replaced with the tag lifetime [1]. I_WR_ is sometimes preferred over I_R_ calculated as Eq. 1.1 because it tends to reflect residency more accurately, for example, not overestimating cases of individuals with few but consecutive detections [124]. This index is also more robust to periods without detections, which can arise for example from difficulties during receiver replacement [124].

##### Continuous time residence

Detections are discrete events or samples of animal movement, which are continuous in time. Continuous time residence (CTR) calculates residency as a continuous event as well, while also considering the effects of small temporal scale biases and movement behaviour [33]. Small scale bias can be caused by environmental noise, obstacles, transmission intervals between signals, and collisions in signal propagation. Accounting for these aspects can prevent, for example, assuming an individual is absent when its presence is masked by external factors. Similarly, at larger temporal scales this method also considers that animals that consistently reside within a particular area can still engage in natural behaviours that lead out of detection range (e.g., diel movement patterns). The goal is to avoid wrongly interpreting an apparent absence as a true absence product of the temporal scale at which residency is being measured [33].

This CTR approach generalizes the method by Ohta and Kakuma, [147] of calculating residency as the continuous presence without absences longer than 24 h around fish aggregating devices. Instead of using 24 h, the period is defined by applying a statistical procedure to the data, also considering the previous knowledge of the researcher(s) [33]. This predefined period is called Maximum Blanking Period (MBP) [183], the maximum time allowed to pass between two consecutive detections before assuming the individual left the area. CTR is then interpreted as a time frame during which an individual was detected without being absent for longer than the MBP [183]. In this interpretation, an ongoing CTR is composed of a series of successive detections separated by a time < MBP and ends when the time between a detection and the next one is > MBP, or when the second detection occurs in a place out of the study area. Both events mark the last detection of an ongoing CTR, and also the first detection in the next CTR (i.e., the detection that ended the CTR) [33]. The MBP should always have a high enough extension to ensure a tag is detected even if a signal collision occurs [183], and the optimal value will depend on the research question and the species. MBP is determined by following a statistical analysis akin to constructing a survival curve, which reflects the probability of the CTR being interrupted by one of the two cases mentioned above. For this, the data is analysed using incremental [1:N] MBP values (N should be higher than the timescale of interest), yielding several CTRs. The objective is to determine the optimal MBP after which the survival curves stabilize. This indicates the timescale at which the confounding elements no longer influence the estimation of residency times and the MBP value that should be used in the calculation of CTR [33]. Some examples of MBP values studies have used can be divided into fine-scale CRTs, which have MBPs from 20 min to one hour and have been used in studies on bull sharks and tunas [78, 87, 139, 184, 197] and large-timescale CRTs with an MBP of 24 h, mostly applied to studies on residency of tunas around fish aggregating devices, e.g. [86, 157, 197]. Such differences highlight the importance of the research question in defining the MBP. Similarly, the time between two consecutive CRTs is defined as large-scale and fine-scale continuous absence time (CAT), respectively [33, 86]. This approach to estimating residence has also applications in social network analysis using automated telemetry systems, e.g., [172]. This residency estimate can be obtained using the log rank statistical test [94], implemented in the survival R package [188], e.g., [9, 190].

#### Pseudo-position estimation from passive acoustic telemetry data

Generally, a successful tag detection is a confirmation that the individual carrying it was within the detection range of a receiver, which is accompanied by a date and time stamp. However, the positions associated with these detections are usually as precise as the detection range of the receiver. Overcoming the lack of fine-scale precision of passive acoustic telemetry is therefore a mandatory first step when using most space use estimators. Locations of greater accuracy also permit an improved view into the position of the tagged animals around critical areas, such as marine protected area borders, or while engaging in behaviours of interest that might have taken place, like spawning, feeding, or refuging. Position estimation methods that use the raw data to obtain more precise position estimates for this end have been developed (triangulation or trilateralization methods), and some will be described in the following section. Before this, it needs to be noted that in the same way a tag can go undetected despite being within detection range, false-positive detections can also happen. These occur when a receiver detects a signal with a tag ID code that was generated from the collision of two or more tags and their frequency, among other reasons, depends on the number of tags simultaneously within range of the receiver [180]. A false detection can produce a code tag ID that is either different or identical to a code tag ID in the study. Cases, where there is no match with a deployed transmitter, are straightforward to detect and exclude, yet these might correspond to a tag of a different study. False code tag IDs identical to one of the tags deployed for the study are harder to detect. False detections do not occur frequently [180], however, ways of filtering them out exist. For example, this can be done by removing single detections within a defined timeframe, using maximum speed estimates of the study animal [100], or custom estimates of precision [182].

##### Center of activity

A center of activity (COA) [179] is a point on a two-dimensional plane whose X and Y coordinates represent the weighted average position of the group of locations, during a given time, used to estimate it [95]. Therefore, rather than a precise location in time, a COA is an average position over a defined period (Δt) selected by the analyst. The detection probability of a transmitter is assumed to increase linearly with proximity to a receiver, as does the number of times the transmitter is detected during Δt. Importantly, as an average position, a COA can represent a location where the animal never actually was. Weighted means are obtained either with an arithmetic (Eq. 3.1) or harmonic approach (Eq. 3.2) and the following variables: number of receivers in the array (n); the number of receptions at the *i*th receiver during Δt (R_i_); the X coordinate (X_i_) and Y coordinate (Y_i_) of the ith receiver. The original paper reports separate equations for each X and Y coordinate, here these should replace the general “coordinate” variable (C) depending on which weighted mean is being calculated. This in turn yields the weighted mean for each coordinate (X̄_Δt_ and Ȳ_Δt_, both represented here by C̄_Δt_).

3.1 $$\overline{C}_{{{\Delta t}}} = \frac{{\mathop \sum \nolimits_{i = 1}^{n} R_{i} C_{i} }}{{\mathop \sum \nolimits_{i = 1}^{n} R_{i} }}$$

3.2 $$\overline{C}_{{{\Delta t}}} = \frac{{\mathop \sum \nolimits_{i = 1}^{n} R_{i} }}{{\left( {\mathop \sum \nolimits_{i = 1}^{n} \left( {\frac{1}{{\mathop \sum \nolimits_{i = 1}^{n} R_{i} C_{i} }}} \right)} \right)}}$$

**Fig. 2:**
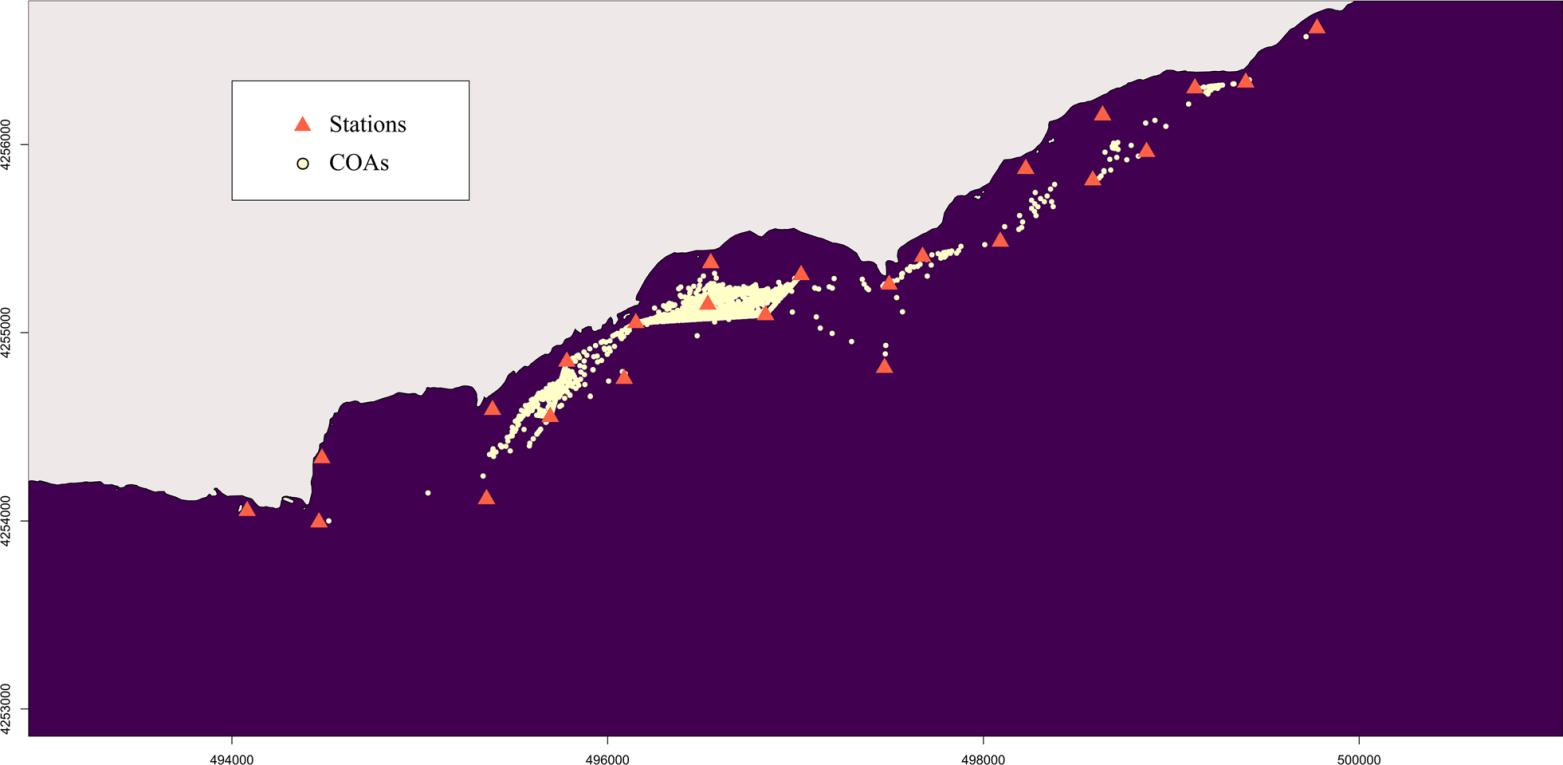
Centers of activity (COAs) obtained for the *Dasyatis pastinaca* data set, using a period of Δt = 30 minutes (each yellow circle) from the sample data set collected by a coastal acoustic receiver array (orange triangles). Land is grey and water is dark blue

This method is sensitive to the selection of Δt, therefore it requires evaluation evaluating before performing calculations, taking into consideration factors such as signal emission frequency, animal activity (rate of movement, speed), and external interferences from topography and vegetation [179]. Too brief windows will not contain sufficient detections to obtain an estimate, while protracted periods can result in excessive movement by the tracked animal, which affects the precision of the activity centre as well as the number of positions generated for analysis [179]. However, vast quantities of locations are normally obtained with acoustic telemetry because tag transmissions delays tend to be about 90–180 s, so a low sample size could potentially become an issue in the event of analyzing small subsets of the total data [23]. Simpfendorfer et al. [179] also recommend that receivers should have overlapping detection ranges, arranged in a grid of triangles, squares, or hexagons. Users should be aware that the spatial configuration of receivers will dramatically affect the COA calculation and further testing of this method is recommended to provide more explicit advice on how to proceed for subsequent analysis. Position estimation using COA is a widely used method in passive acoustic telemetry. Users may wish to use pseudo-positions to replace the need for actual positions for methods such as kernel density estimation that require them. Calculation of the COA can be obtained using R-packages like V-Track’s Animal Tracking Toolbox (ATT) [32, 192], however, users can easily calculate this metric by simply grouping detections by individual ID and a time window and calculating the mean longitude and latitude. Figure 2 illustrates the 30-min COAs obtained from the *Dasyatis pastinaca* sample data set that will be used in the paper.

Hedger et al. [96] evaluated the local polynomial regression or Freidman’s SuperSmoother [81] as a non-parametric alternative to the weighted mean COA. An advanced method of calculating COA has been developed by Winton et al. [205] using the Bayesian spatial point process (SPP) model that also allows accounting for the variation of detection probability over time, yet the authors acknowledge that the computational expense of the method may make it inaccessible to many users. Detections of a tagged animal are viewed as samples of an underlying spatial process that depends on (therefore is biased by) the observation process, which is composed of the position and detection range of the receivers used to detect the tags. Receiver- and time-related variations in detection probability can be included in the SPP model, by integrating data from stationary test tags in the study area. Including this information improves the model, as it can reveal otherwise overlooked fine-scale movements. These characteristics make it a more computationally demanding process than the mean weighted COA, however, it is comparatively less biased, especially when including data on variation in detection probability. Detections that are only recorded by peripheral receivers in the array (i.e., from individuals present in the area but not within the array) are also used, and lower errors are obtained for position estimations in these areas. COAs can be estimated with the SPP model using the R package TelemetrySpace [205]. An important difference between this method of estimating pseudo-positions and the triangulation-based methods in the following section is the area where a calculated pseudo-position/position can be found. COA-based estimation can only place a calculated pseudo-position within the area confined by the array, while triangulation-based methods can allocate them to areas out of receiver range, although with lower precision [182].

#### Triangulation or trilateralization

##### Proprietary positioning systems

###### *Vemco (now Innovasea) positioning system*

The Vemco positioning system (VPS) is a fine-scale positioning system that estimates positions by using omnidirectional receivers and fixed synchronizing transmitters, or sync tags [62, 182]. Such high-resolution tracking systems have allowed greater detail in residency and home range estimation, and also to venture into other areas such as behavioural studies, as shown by [150].

Synchronization tags that transmit at fixed intervals are placed at known locations to help synchronize receivers and are deployed either over each receiver [162, 182] or in a way that one sync tag’s detection range encompasses receivers in the aforementioned groups of three [62]. Positions are estimated based on hyperbolic positioning or time difference-of-arrival (TDOA). The difference in detection time between pairs of receivers indicates which receiver detected it first, and how much time passed until it was detected by the second receiver, which is used to calculate the distance of the tag to each receiver. Distance difference and receiver positions are used to obtain an approximate location for the transmission in a hyperbolic position system. VPS calculates one basic position for every possible group of three receivers that detected a given transmission, which is then combined to calculate a synthesized position [182]. Positional error is expressed as Horizontal Position Error (HPE), a relative unitless estimate of error sensitivity used to retain the highest quality estimated positions [62, 167], which is not comparable across studies because calibration is specific to each study [182]. Higher HPE means a calculated position is more sensitive to measurement errors, hence, a lower HPE is preferred [162, 182]. The accuracy estimation of calculated positions requires stationary transmitters at known locations to compare with [182]. Even though high-resolution of positions are obtained with systems like VPS or PinPoint, the data may still need to be filtered before analyzing it as suggested by some authors (e.g., [130, 167]. For example, filtering VPS data by the HPE can greatly reduce the positioning error in the data set [130, 167].

More recently, Innovasea introduced high residence (HR) tags and receivers, which can be combined into an HR-VPS system that operates at 180 kHz [91], allowing to simultaneously monitor a higher number of tags. Such characteristics are ideal when many animals aggregate because of migratory, reproductive, feeding, or geographic reasons [91]. However, it must be noted that frequencies above 100 kHz are greatly attenuated by salt water [3], which affects transmitter efficiency. Vemco/Innovasea developed the software Vemco user environment (VUE),^1^ which centralized many tasks. Among these, it is used to collect, organize, and visualize the data, also allowing to run some initial analyses. VUE is also used for tasks like receiver clock synchronization and memory clearing. From here, data sets can be exported to be used in other programs.

###### Thelma pinpoint positioning system

PinPoint^2^ is a service provided by Thelma Biotel (Trondheim, Norway) that can operate in two and three dimensions, depending on whether the tags are equipped with depth sensors. For this method, receivers in the array are ideally organized in equilateral triangles to maintain the same distance and angle between neighbouring receivers. Thelma runs a service that provides the best deployment configuration of the receivers in the array to obtain the best coverage of the study area.

Like other high precision positioning systems, PinPoint uses time difference of arrival to calculate fish positions, with synchronized clocks and temperature sensors in the receivers. Error sensitivity in the calculation of positions (horizontal dilution of precision, HDOP) is estimated by Thelma (as Innovasea’s HPE). This is done by placing a grid of points around the receiver array, for each of which the signal travel time to reach each receiver is calculated. Then a random time error is added to each position, yielding simulated travel times which are replicated several times with a new random error for every grid point. The deviation between these calculated positions and the known position is then referred to as HDOP. Higher HDOP values indicate higher error sensitivity [140].

Thelma also developed the software ComPort^3^ to upload the data, visualize it in several ways, and do preliminary explorations. This software can also be used to configure receivers and manage the data in an SQLite database, allowing to clean and filter it, for example, to remove false detections before analysing it with software such as R. Filtering can be done using the HDOP, commonly defined as being proportional to a location error’s standard deviation, with higher HDOP values indicating greater variance [134].

##### Open-source positioning systems

###### *Lotek code division multiple access*

The Lotek MAP acoustic telemetry system is based on the code division multiple access (CDMA) technology, used to enhance GPS precision and to provide many users simultaneous use of a cellular network also used in acoustic telemetry [43]. Central receivers are used to monitor, clock-synchronize, and store the information of additional hydrophones. CDMA systems also achieve higher sampling rates (faster pulse bursts), better code discrimination in cases of high noise environments, multipath signals (detections that did not travel in a straight line between tag and receiver) or overlapping signals, and lower signal-to-noise ratio threshold, meaning signal power does not need to be much stronger than noise power. Moreover, signal output appears to be less affected by distance compared to other tags [17]. These characteristics allow to simultaneously monitor a higher number of transmitters compared to pulse-position coding [40]. CDMA telemetry can provide data at a broad arrange of spatial (across a study area to sub-meter) and temporal scales (seasonal to seconds) [93]. For example, this suits studies of fish activity near boundaries, like in an existing or proposed reserve [40], giving the method applicability in conservation and management. This system uses hyperbolic triangulation to position tags, which requires a theoretical minimum of three receivers to simultaneously detect a tag to calculate a two-dimensional location [144].

More control and insight into the data filtering process and quality checks are possible as these are done by the researcher using the company’s software to manage information, estimate transmitter positions, and evaluate performance, unlike with VPS [17]. This process filters out position estimates that fall in areas known to be inaccessible to the animal (e.g., a fish on land), that arise from impossible movements like excessively fast speeds, or are of insufficient precision. This is performed using two measures, dilution-of-precision (DOP) and reliability index (RI). DOP predicts precision levels for a given receiver array design and maps the results. On the other hand, the RI is indicative of the effective contribution of all receivers to a calculated position [144]. During the study design, mathematical modelling, DOP, and RI are used to maximize the position estimate precision, allowing to objectively predict data quality and select an appropriate array design [144]. Such high-frequency acoustic systems are generally thought to be less efficient in the marine environment compared to fresh water, but field tests have proven them to be useful when studying species of small home range size [6].

###### Yet another positioning solver

Many manufacturers obtain estimate positions and their associated errors using methods that are commonly unknown to the researchers, resulting in lower control over the analysis process and hindered comparability across studies [10, 11]. Yet another positioning solver, or YAPS [11], is one of the newest fine-scale positioning estimators and uses time of arrival calculations to position tangs in a receiver grid. The implementation seeks to maximize the utilization of data in a transparent and open source way that applies to all acoustic telemetry brands and indeed to any acoustic signal that can be multilateralized among stations [10, 11]. An associated R package, *yaps* [10], is available on CRAN and in ongoing development on GitHub.^4^ The workhorse of YAPS is the synchronization model, which relies on the user to create a list of data frames including the receiver locations with corresponding sync tags (sync tags are assumed to be attached to each receiver) and sync tag detection data. The synchronization tag detections within the network will determine the suitability of the design for triangulating animal positions in the next step. The sync model residuals can be evaluated by the user and tuned to optimize performance. The synchronization model is then applied to the remainder of the data such that receivers are operating on the same clock and times of arrival at receivers are exact.

Once the receivers are synchronized, the analyst can generate time of arrival estimates and fit the yaps function to the data, typically in chunks separated into one individual per day. Instead of using time-differences of arrival (TDOA) to obtain position estimates as other position estimators (*e.g.,* VPS), YAPS directly uses each location’s time of arrival (TOA) at each receiver, which allows taking fuller advantage of the data. YAPS does not require a signal to be detected by at least three receivers, avoiding information loss by discarding detections. Additionally, instead of operating like a point-by-point positioning model that calculates each position separately, YAPS calculates tracks directly, fitting a movement model to the raw detection data [11]. A state-space model is applied to the TOA data, composed of 1) a process model and 2) an observational model. The process model describes the system’s dynamics and the transmitters’ coordinates over time, assuming a random walk between transmissions. Estimations are performed for transmission time (i.e., time of signal emission), transmitter coordinates, and speed of sound [11]. The latter, and arrival time at a receiver, are used to calculate the time of signal emission. The observational model relates unobserved processes to the data. It calculates distances between the transmitter position at the time of signal emission and the position of all receivers, relating the observed time of the signal. Finally, residuals between observed and predicted times of transmission arrival are defined, accounting for detections from multipath propagation. A Maximum Likelihood analysis selects the track with the lowest error [11]. An advantage of YAPS is that the inclusion of a movement model fitted to the raw TOA data yields biologically sound position estimates [11]. Compared to VPS, YAPS has been shown to yield more position estimates per track, to better correct reflected signals and model fish behaviour when the fish are not within the acoustic array but still within range, and to be more robust in highly reflective environments [199]. However, since a movement model is directly applied to the raw data, YAPS requires a minimum number of total transmissions, unlike point-by-point estimators [11, 198]. YAPS also requires receivers to be synchronized before analysing the data, which is simultaneously done for all receivers, allowing to reduce the amount of error compared to approaches like sequential synchronization [10]. A critical factor for the performance of YAPS is knowing when the next signal will be emitted, and constant intervals or known random intervals significantly improve the accuracy of position estimates over unknown random intervals, as some manufacturers operate [198]. When ping sequences are not known, a random burst interval is set as default. YAPS should be iterated so that each fish day is run multiple times with the best fit, determined by the $obj value returned from the output. The user may determine how many runs are optimal, but 5–20 could be sufficient, with up to 50 for difficult tracks (Baktoft, personal communication). Poor data may never fit and will have to be discarded. Despite its advantages, YAPS is time-demanding and requires considerable work and expertise on the part of the analyst along with access to sufficient computing resources [199]. Although guidelines have been developed [10], receiver array synchronization has proven to be challenging for many users [10, 199].

### Accuracy

The accuracy of the position estimates obtained with triangulation-based systems is much higher than that of COA-based methods. A table compiling the accuracy of the former is available in the supplementary material of [128].

#### Geometric, hull-based estimators

##### Minimum convex polygon

**Fig. 3:**
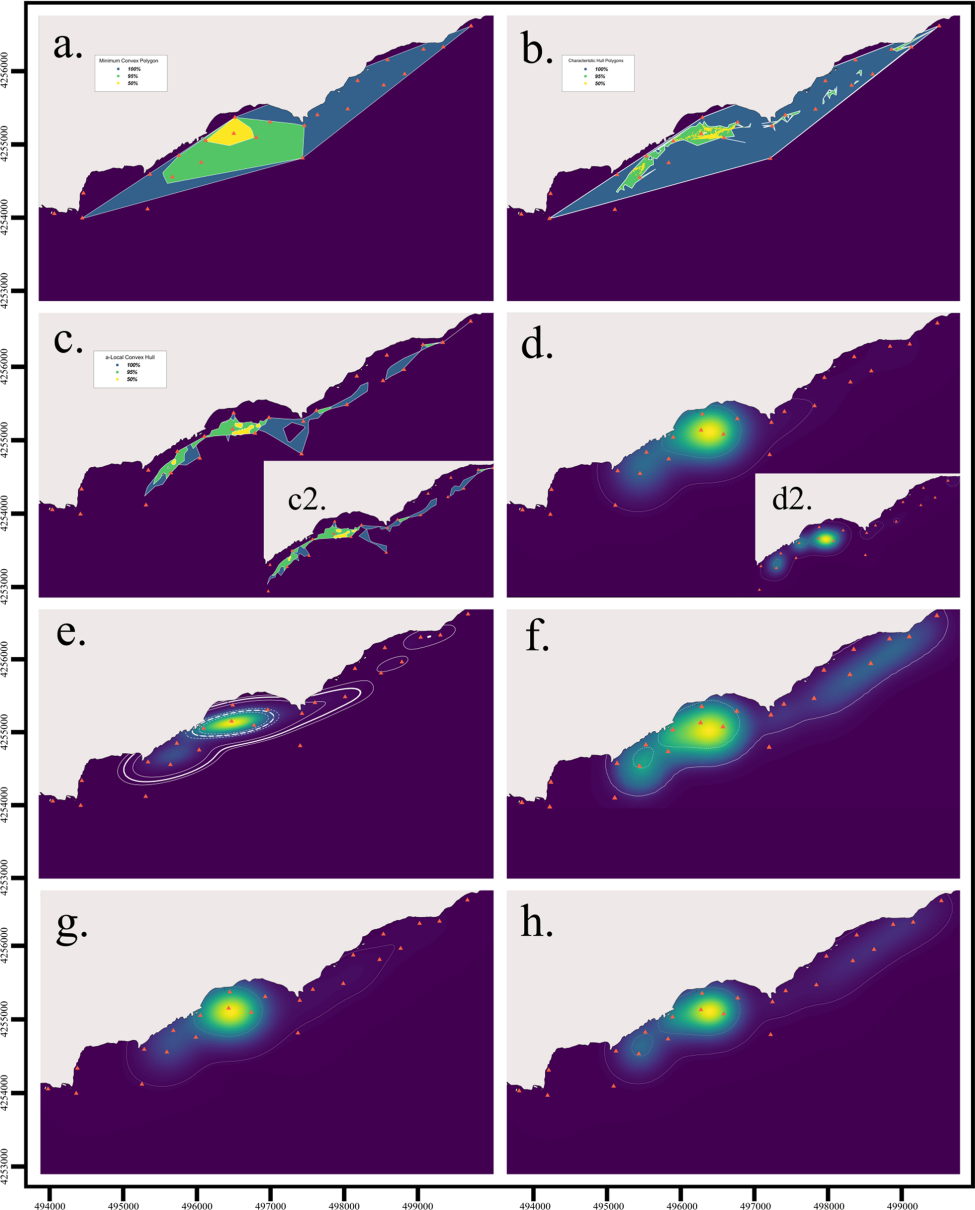
Examples of space use estimation with some of the methods described in this paper using the *Dasyatis pastinaca* data set. Land is grey and water is dark blue. **a** Minimum Convex Polygon at 100% (blue), 95% (green) and 50% (yellow) isopleths; **b** Characteristic Hull Polygons at the 100% (blue), 95% (green) and 50% (yellow) isopleths. Note the 100% CHP is equal to the 100% MCP. The estimations at lower percentage levels (95% and 50%) differ from their MCP counterparts because of how each method constructs polygons. Discontinuous area estimates and highly irregular shapes can occur depending on the characteristics of the real-life data used; **c** a-Local Convex Hull using a value of a = 600 m, with the 100% isopleth in blue, 95% isopleth in green and 50% in yellow. The inserted picture **c2** shows another example using a value of a = 1000 m; **d** Kernel Utilisation Distribution estimation method using sample data at the 95% (solid white line) and 50% (dotted white line) isopleths using a fixed bandwidth of h=250. The inserted picture **d2** shows another example using reference bandwidth href = 116 to highlight the effect of bandwidth selection; **e** Optimally weighted AKDE_C_ (wAKDE_C_) range estimation with confidence intervals using an anisotropic OU movement model. The 50% core area (bold dashed line), 95% home range (bold solid line) and their respective confidence intervals (thinner dotted and solid lines) are shown; **f** Brownian Bridge Movement Model at the 95% (solid white line) and 50% (dotted white line) isopleths, using a location error of 250 m; **g** Biased Random Bridge kernel method at the 95% (solid white line) and 50% (dotted white line) isopleths, using a diffusion parameter of D = 4.44, L_min_ of 50 m, a minimum smoothing parameter of h=250; **h** Dynamic Brownian Bridge Movement Model at the 95% (solid white line) and 50% (dotted white line) isopleths, using a location error of 250 m, margin size *m* = 11, and window size *w* = 31. Images **a**–**d** and **g** were created using the R-package adehabitatHR, **e** using the R-package ctmm following the R script provided by Silva et al., [178], **f** using the Animal Tracking Toolbox in the R-package V-Track, and **h** using the R-package move. All images were posteriorly edited for publication

Minimum Convex Polygons (MCP) [135] are among the first home range estimation methods, which represent a two-dimensional maximum area estimate for a group of locations obtained by tracing the smallest polygon possible using only the exterior points and interior angles under 180° (Fig. 3a). Polygons may be drawn using raw detections that represent the receiver coordinates or based on calculated pseudo-positions or positions. Because of their simplicity, MCPs are fast to compute and have been widely used for decades, which has provided much comparative material [120, 145]. Polygons, and MCPs in particular, are sometimes used to estimate the maximum area used by an animal [145]. The International Union for the Conservation of Nature currently applies it as a proxy to calculate the maximum extent of occurrence, a measure of extinction risk [104]. Using MCP in these assessments has been thought to ensure there is consistency among comparisons [111], although alternative methods that deal better with range discontinuities and are not biased with increasing sample size have been proposed, such as α-hulls [25]. The polygon-based approach has been said to be too simple and fails to correctly characterize and predict the distribution of species, e.g. ([158, 159 while others support their use, especially in data-poor situations, e.g. [160].

Yet, MCPs present several drawbacks [89, 163] and some even advise against their use [120]. Only considering the outermost locations dismisses all internal data points and the information these might convey, and an even use of the area within the polygon is assumed [163]. This precludes detecting heterogeneities in animal movement like preferred and unused areas, and boundaries to movement. MCPs are sensitive to habitat shape, location error, and distribution of sampling effort in space and time [25], extreme location points from sporadic forays into adjacent areas [89], and non-compliance with location independence, which results in the underestimation of home range [186]. In acoustic telemetry, MCP dimensions will be limited by the array design, as areas excluded from receiver coverage cannot be included in the polygon, which may dramatically misestimate the dimensions of the polygon in a way that misinforms the ecology of the animal. Some of these shortcomings can be addressed in a few ways. Constructing MCPs using 95% of the data that form the smallest polygon reduces the inclusion of rarely or not visited areas and accounts for the sensitivity to extreme locations [67, 163]. Creating monthly or seasonal polygons can be a more biologically-sound approximation to space use [89]. Finally, since MCPs are prone to sample size bias, communicating details about this in the study can help to produce more comparable results [22, 25, 67, 145, 207].

##### Characteristic hull polygons

The characteristic hull polygons (CHPs) method [52] is a hull construction approximation based on the characteristic shapes algorithm [57]. CHPs are obtained by creating triangles by Delaunay triangulation of neighbouring points, which favours the construction of more regularly shaped triangles. Home range estimates are obtained by removing the triangles with the largest perimeters and retraining the 95% of the smallest triangles. Other features like area can be used as a sorting criterion, but perimeter allows to eliminate the slenderer triangles with exceptionally acute angles that normally form at the boundaries of the used area. An MCP equivalent is obtained if no triangles are removed [52]. Point distribution of the data is better represented using Delauney triangles than with MCPs, as the former method can build non-convex hulls and more complex shapes [52, 57] (Fig. 3b.). CHPs can also have “holes” and be composed of disjoint polygons, which can accommodate distribution patterns of animals that avoid certain areas. CHP are fairly robust to sample size variations and to inhomogeneous point distributions [51] and do not overestimate areas of use like MCP [52, 148]. However, they perform relatively worse when home ranges have a concave, or convex shape compared to linear, disjoint or perforated, but this could be related to the process of removing triangles [52]. Despite its advantages, CHP are not as studied and frequently used as MCP [52, 165]. The Delaunay triangulation required for producing CHP can be implemented in many GIS softwares [52, 57], such as standard functions in ArcGIS^5^ (ESRI, Inc., requires subscription purchase) e.g., [51, 148] and also QGIS^6^ (free and open-source). In the former, the hot spot analysis with rendering spatial statistical tool can be used as a more objective way of selecting triangles [112].

##### Local convex hull (LoCoH) methods

The k-LoCoH or k-nearest neighbour convex hull (k-NNCH) is an extension of the MCP in which space use is estimated by constructing local convex hulls around each point using its *k*-1 closest neighbours, akin to small MCPs. The obtained hulls are then ordered from smallest to largest and merged to create area isopleths that contain a proportion of the data (e.g., 50%, 95%) and utilization distributions (UDs) from the proportion of points contained in each local convex hull. This approximation allows to better account for boundaries produced by geographic features or other factors [84], but can take much longer to compute, increasing exponentially with the size of the data (adehabitatHR manual, Calenge [31]. Selecting the number of nearest neighbours (*k*) is user-defined and is a crucial step that follows the minimum spurious hole covering (MSHC) rule. Low *k* values generate coverings that contain “holes” that disappear as *k* increases. In real landscapes, such “holes” (or unused areas) can be produced by features like cliffs, mountains, lakes, and water edges, which represent restrictions to movement. Following this rule, the smallest *k*-value that produces a convex hull reconstruction with a shape that matches the study area of known topology (i.e., known location of “holes”) is selected. If the topology is unknown, large features can be identified to guide this process [84]. Because LoCoH draws the kernel shape directly from the data, it tends to perform better close to boundaries than MCP and kernel-based methods [84, 123, 170]. By not including unused areas, LoCoH methods are less prone to type I errors (to include unused areas in the estimate) than the aforementioned methods, although, in turn, this can increase the risk of type II errors (exclusion of used areas) [123, 168].

Two variations of k-LoCoH exist, the fixed radius r-LoCoH and the adaptive a-LoCoH [83, 84] (Fig. 3c, c2.). They differ in the calculation process and sorting method of local hulls, but overall operate in the same way [83]. The r-LoCoH uses all points within a radius *r* from a root point, resulting in hulls of similar size but may differ in the contained number of points. This is used to sort circles in descending order (hull area as the second criterion). On the other hand, a-LoCoH creates circles of variable size around root points of radius size depending on the cumulative distance from the root point to its nearest neighbours until it is equal or as close to the defined maximum cumulative distance (*a*). This way areas with higher point density (or of higher use) will have more points at shorter distances from the root point, resulting in smaller convex hulls, and vice versa. This can be used to identify range edges where points can concentrate, like shorelines or cliff edges [83]. If the topology of the study area is known and unused areas can be identified, the MSHC rule can be followed to select *r* and *a* [83]. Of the three, a-LoCoH is the best performing method [83, 170], as it is more reliable in the absence of topological information, while r-LoCoH usually performs the poorest.

However, because movement information is ignored, UD estimates can be of lower resolution and present home range boundary biases compared to methods that include it [14, 15]. This drawback is addressed by the Time Local Convex Hull (T-LoCoH) [127], which integrates both temporal and spatial dimensions to serially correlates points in the construction of local hulls. The inclusion of timestamps during nearest neighbour selection and local hull sorting allows separating spatially proximate but temporally distant locations and local hulls. The nearest neighbour selection follows the time-scaled distance (TSD) metric, which transforms time into a third distance axis using a maximum theoretical velocity (v_max_) and the dimensionless scaling parameter *s*. v_max_ can be drawn from biological studies, statistical models [125], or the maximum segment velocity in the data [127]. The elapsed time between two consecutive locations and v_max_ is used to obtain a theoretical maximum traveled distance [127]. The parameter *s* controls the importance of time in the modelling of space use: a higher value gives time more weight and setting *s* to 0 renders it equivalent to k-LoCoH. The value of *s* is subjective, yet it can be determined following guidelines [127], and a more objective approach based on cross-validation has also been proposed to select both *s* and *k* [49]. Compared to hulls created without considering time, isopleths from TSD hulls better identify temporal changes in movement patterns and spatially overlapping but temporally differentiated resources. Unlike the Brownian bridge-based methods (covered later), which integrate time with a segment-based approach, TSD is calculated for all possible pairs of points [127]. As a result, points that are close in space, yet occurred in separate time frames are “pushed” away by this time-distance axis. In turn, the obtained local hulls share similar traits, as they also are local in space and time and the boundaries of resource patches that spatially overlap but are used at different times are preserved [127]. Local hulls created using the TSD method can be used to estimate the level of directionality in movement and time-use. The type of movement (*e.g.,* more sinuous, or linear) can be estimated by assessing hull elongation metrics to identify possible transit areas or of low resource value, while calculating time spent in an area (duration of use) and rate of revisitation an area has can be used to infer the type of use an animal gives to it [127]. Finally, local hulls can be sorted following one of several hull metrics (e.g. area, perimeter/area ratio, revisitation rate, duration of visit, date of root point) and combined to obtain isopleths that highlight different types of information depending on the objectives of the analysis [127].

#### Density distribution probabilistic estimators

##### Kernel utilization distribution

Before describing this method, the concepts of Utilization Distribution (UD) and bandwidth or smoothing factor (*h*) need to be defined.

###### Utilization distribution

This concept refers to the use of the observed location data points of an animal to create a two-dimensional relative frequency distribution in an area over a specific time [196]. UDs, therefore, allow the description of space use in terms of a probabilistic model to represent the probable location of an animal on a plane, which is used to estimate metrics such as home range [196, 206]. UD is directly influenced by the smoothing factor or bandwidth used in kernel methods [206].

###### Smoothing factor or bandwidth

It is the standard deviation of the kernel, the extent to which a location is allowed to influence the home range estimation or the distance over which it is allowed to influence the total density estimate. It is central to kernel analyses, as it can have significant effects on results [116, 163, 171], 200]). Its (1) value and the (2) number of bandwidths are user-defined.

- (1) Value: A higher smoothing factor (*h*) widens the UD over each data point and allows more distant points to have greater influence, increasing the overall home range size [163, 171, 206]. Higher *h* also smooths out sampling errors (for instance, related to telemetry error) and eliminates details at finer scales, retaining only the most notorious features [163, 171, 206]. Contrarily, a smaller *h* provides greater detail at small scales, yet tends to be more sensitive to measurement error [163, 206]. No universal method to determine the optimal *h* value exists [206], but several ways exist to determine it, like least-square cross-validation, reference bandwidth, ad-hoc choice of *h*, its variation *h*_ad hoc_, direct plug-in, and solve the-equation [60, 116, 206]. In some instances, bandwidth is selected manually based on information about detection range in the array [129]. Bandwidth can also be altered if space use estimates result smaller, separate isopleths, but having them form a single, continuous area makes more biological sense [116],Wand and Jones, 1995). This can be done using an ad-hoc smoothing parameter, which seeks the reference bandwidth value just before space use fractures or gaps appear [16, 116].
- (2) *h* can either be fixed (one value for *h* is used) or vary according to point density. The former is also called global bandwidth and will result in a fixed-kernel analysis, while the latter is also known as local bandwidth and results in an adaptive-kernel analysis [206]. Kie [116] presents a detailed analysis of how bandwidth selection, sample size, and fixed- and adaptive-kernel estimates interact. The use of a fixed or a variable kernel has long been debated, yet studies have shown that the selection of one over the other does not influence bias and type I and II errors as much as the choice of h [116].

###### Kernel utilization distribution

Kernel utilization distribution (KUD) [206] is a non-parametric statistical method regarded as one of the most popular and best-known estimators in use [120, 163]. KUD calculates the area of probability of finding an individual, similar to sampling a distribution of occurrence, by placing a kernel or probability density function (PDF) over each data point. Kernels can be visualized as a three-dimensional “hill” [206], whose shape and width (*h*) are user-defined [163, 206]. Kernel shape does not influence the results as significantly as the smoothing factor [61]. Once parameters are defined and kernels are in place, a grid is positioned over the area to calculate estimates of density by averaging the densities of all kernels that overlap at each grid intersection [206] (Fig. 3d, d2.).

KUD is a straightforward method to estimate home range and has much supporting statistical literature, yet it presents important caveats. Locations are assumed to be uncorrelated, or independent of each other and identically distributed (IID) [207]. While a sufficiently spaced-out sampling complies with this, it is unlikely to be the case with the high-rate samplings of acoustic telemetry. The inherent autocorrelation of movement data has been regarded as a valuable source of biologically relevant information [46], and not meeting this assumption can result in biased results like underestimated home range sizes [76, 186]. Since the PDF is applied in all directions around a point, areas that are not part of the animal’s home range can be included, like areas around narrow trails whose dimensions greatly differ from the selected bandwidth [164] or over impenetrable barriers. Other bias sources are sample size [97], especially overestimation at low effective sample size [85], and point pattern shape [53]. KUD is implemented in packages like adehabitatHR [31], ks [36], and amt [174].

##### Autocorrelated kernel density estimation

The group of autocorrelated kernel density estimation (AKDE) methods is the generalized version of the Gaussian reference function KDE which addresses commonly encountered biases during space use estimation. Nowadays three methods exist, the AKDE [76], area-corrected AKDE (AKDE_C_) [72], and optimally weighted AKDE_C_ (wAKDE_C_) [77]. AKDEs incorporate autocorrelation into range estimation, tackling KDE’s assumption of IID data and space use underestimation. This and the associated confidence interval that is provided have improved accuracy in comparison [146]. Location independence, and avoiding underestimating home ranges, requires using a sampling periodicity that is above the autocorrelation timescale, or at least equal to the home range crossing time (the time it takes for an individual to cross the linear extent of its home range), which can vary greatly by species [76]. This means that given the same number of locations in one autocorrelated and one uncorrelated data set, the former contains an overestimated sample and less positional information. To have similar positional information and be as informative, autocorrelated data would need to be larger and span for a much longer period [76]. Moreover, autocorrelation becomes stronger with more frequent sampling [186], which is mostly the case with acoustic telemetry. To explicitly include autocorrelation in home range estimation, AKDE requires the selection of a movement model that includes autocorrelation in the analysis and then uses a Gaussian reference function to calculate bandwidth. Currently, available movement models are an IID process with uncorrelated locations and velocities, Ornstein–Uhlenbeck (OU) process with correlated locations and uncorrelated velocities [194], and an Ornstein–Uhlenbeck Foraging (OUF) process of correlated locations and velocities [73, 74].

AKDE’s use of the Gaussian reference function creates a positive bias in area estimation, which is adjusted by the area-corrected AKDE (AKDE_C_) ([72]. It calculates the level of oversmoothing and corrects it to create improved area estimates, especially at low effective sample sizes where positive bias is strongest. As a result, the contour of the calculated areas is drawn towards where the data points are higher in density under all autocorrelation movement models [72].

More recently, [77] introduced the optimally weighted AKDE_C_ (wAKDE_C_) which optimizes estimates by correcting time-related sampling biases, i.e., irregularly collected or missing data (Fig. 3e.). Such issues can arise from equipment malfunction, signal loss related to habitat interference (common in aquatic environments), or behaviour, among others. This way the importance of the areas an individual visits is appropriately levelled by upweighting under-sampled areas and downweighting over-sampled areas [77]. Additionally, similar to Fleming and Calabrese, [72], this approximation improves home range estimations from evenly sampled but small effective sample size data [77]. AKDE is included in the R package continuous-time movement modeling (ctmm) [29] and in the point-and-click graphical interface *ctmmweb* [30]. Guidelines for the use of these estimators and the correction of the biases here described are compiled into a document that includes an R script [178].^7^

##### State-space models

Essentially, a state-space model (SSM) analyses movement data and integrates error correction, the calculation of metrics, and statistical analysis by combining (1) an observational model to statistically describe the sampling process and (2) a process or movement model, which relates to the description of the dynamics of movement in space and time. As a Hidden Markov Model with discrete hidden behavioural states, this method is useful to use movement data to infer behavioural modes and estimate the probability of being in a given behavioural state [153].

The Ornstein–Uhlenbeck SSM (OU-SSM) [156] is a SSM adapted to estimate home range using acoustic telemetry data in which the observation model describes the probability of a receiver detecting a tagged animal. At a given time, each station in a receiver array will either record the presence or absence of a tracked individual, information which is used by the detection function to model positions and compared to other non-mechanistic methods [96, 179] presents improvements in the estimation process [156]. This function computes the most likely position of the animal at a given time by combining the presence/absence information from all receivers in the array, for which changes in detection probability over time can be accounted for [156]. As a function of distance, at the time of successful detection, the likelihood of an animal’s position is higher closer to the receiver that detected it. The detection function also incorporates positional information on undetected individuals, assuming they are more likely to be found in areas farther from the receiver [156].

Changes in detection probability over time produced by environmental interference, for example, day/night cycle, seasons, or temperature, can also be integrated into the model by collecting reference data at the study site. Not accounting for this can lead to misinterpretation of results (for example unaccounted diel change in detection range can be wrongly interpreted as the animal leaving during what is a period of reduced detection capability), however, this is not straightforward to incorporate [156].

Additionally, the movement model characterizes the dynamics of the tracked animal through space and time. The OU-SSM model implements a OU process [18], a modification of a continuous random walk model. It considers a fixed point of attraction that introduces a movement bias towards it, which can biologically be considered as the home range centre and makes it suitable for studying species that are normally monitored with acoustic arrays [156]. Furthermore, the SSM optimizes the position estimation at a given time by taking the information provided by other points that are proximate in time, obtaining a range of possible positions given a maximum speed. The SSM is applied as a spatial hidden Markov Model [155, 156], with discrete hidden states (in this case, locations) generated by an unobserved Markov process. In a Markov process, the probability of a future state (the next location) is only dependent on its current and past state. This stochastic process can be of first-order, depending only on the current state, or of higher order, depending on the previous state or more [153].

Later a Bayesian SSM (B-SSM) was introduced [5]. The observational model calculates the detection probabilities based on the distance between animals and receivers, also considering the effect of environmental factors [5]. The process model or movement model is based on the OU-SSM [156] and, rather than an upgrade, is proposed as a Bayesian alternative to the frequentist approach of the OU-SSM [5]. This combines existing knowledge (as prior probabilities) and data-derived information through maximum likelihood, to obtain the posterior distribution of the movement parameters and movement path [5]. Positions are estimated by considering the previous position and the following movement parameters: position of the home range center at a given time (r^H^), exploration rate of the home range in min^−1^ (*k*), and size of the circular home range (radius, *r*). The Bayesian SSM is computationally more demanding than frequentist SSMs, however, the R package *Template Model Builder* [4], fits SSM models to movement data and can compensate for this [5]. Additionally, the supporting information of the manuscript includes code that can be used in R to estimate movement parameters (home range behaviour) and positions with acoustic tracking data [5].

##### Brownian bridge movement model

Brownian bridge movement model (BBMM, Fig. 3f.) interprets data as a collection of consecutive, known locations and models a time-structured path to reconstruct the expected track the animal traversed [24, 102]. By accounting for autocorrelation, BBMM can provide biologically more meaningful results compared to KUD [102] and is less sensitive to irregular sampling because it considers time differences between locations [119]. Animal tracks are commonly modelled with a random walk, which is a stochastic process of random and discrete steps (i.e., composed of integers) taken on a space. Brownian bridges are based on diffusion-based Brownian motion, which is like a random walk but occurs on a space and time continuum and is conditioned at the beginning and the end by a pair of known, consecutive locations [102]. Path modelling between successive locations is done using a Brownian bridge function that consists of a probability of occurrence calculated along the path traced by the animal [102]. The probability distribution spreads in the direction of movement instead of in every direction as in KUD. This way using time-ordered location points allows to link areas of frequent use, while areas that the animal does not use can be excluded [102]. The model includes the animal’s mobility as the Brownian motion variance parameter (*σ*^*2*^_*m*_), which is related to the animal’s speed and represents the area an animal could use between locations. Using a single value for this parameter simplifies calculations, however, allowing it to vary would better reflect different behaviours of an animal, as the parameter relates to its movement which can naturally vary over time [102]. The location data of each animal is used to independently calculate one *σ*^*2*^_*m*_ value for their respective track [102].

The BBMM’s robustness in identifying movement paths is reduced with increasing time intervals between locations, as the assumption of random movement is weakened by increasing location error in the data and mobility (i.e., behaviour) [102]. Additionally, the assumption of purely diffusive movements might not effectively estimate home range and habitat preferences in all cases because it dismisses biologically meaningful information that might be behind changes in movement patterns [14]. Diffusive movement best suits animals that continuously move in a constant environment, i.e., with no home range and with randomly distributed resources [14]. Therefore, the movement of an animal between two locations very distant in time may be more accurately modelled as a biased random walk rather than a purely diffusive random walk such as that of the BBMM [102]. Importantly, because the probability distribution here computed is constrained by these pairs of locations at both ends, the BBMM and the next two Brownian bridge models are occurrence or trajectory estimators rather than home range estimators in the strict sense [75, 76]. Therefore, the path of the animal during the monitoring period is modelled, which does not involve a range prediction into the future [76].

##### Biased random bridge kernel method

The main difference between the advective–diffusive or biased random bridge kernel method (BRB) and the previously described BBMM is that the latter purely includes diffusive movements, making it less suitable to estimate home ranges [14]. The BBMM also assumes a constant animal mobility value, which dismisses behavioural changes in movement, for example, changes in speed, direction, and permanence in preferred areas [14]. As opposed to the BBMM, the advective component is allowed to vary between bridges but must be constant within each one [14].

The BRB uses the movement-based kernel density estimation (MKDE) to calculate BRB-based UDs [15], which allow flexibility in the animal’s movement and more accurately estimate home range. This method focuses on active UDs and not on global UDs (which include resting phases) by assuming that space use intensity is proportional to activity time spent. This also results in differences regarding the way the smoothing extends over space. KDE UDs extend in all directions around a location point, while MKDE’s active UDs distribute probability density along a track between pairs of observed locations by interpolating positions between them (creating new points between two observed ones) [15]. To ensure that the animal was between the two observed points and not elsewhere in its home range during the time elapsed between them, an upper recording time limit (*Tmax*) is used to identify data points that are too distant in time to be safely considered correlated, which get no locations interpolated between them and are filtered out for the calculations of BRB [14, 15]. These positions of high uncertainty would be included by the BBMM [14]. *Tmax* is used to apply a variable smoothing factor to all observed and interpolated locations, assigning the lowest smoothing factor value (*h*_*min*_) to the recorded positions and the highest (*h*_*max*_) to the interpolated position found at the midpoint of the largest segment (i.e., of size *Tmax*). Therefore, the longer the time between an interpolated and a recorded position, the higher *h* or uncertainty of the position. Habitat-specific diffusion coefficients (*D*_*H*_) can be identified by calculating the diffusion coefficient of all track segments in the same habitat type. This can be included in the MKDE by assigning habitat-specific *h*_*max*_ and allowing to differentiate, for example, preferred habitat types from others the tracked animal traverses faster. The MKDE also addresses the boundary biases typically found in traditional kernel density estimators, by modeling boundaries as contiguous straight segments and correcting them via boundary-based coordinate transformations [15]. BRB is available in the R package adehabitatHR [31] (Fig. 3g).

##### Dynamic Brownian bridge movement model

The Dynamic Brownian bridge movement model (dBBMM) [119] extends the BBMM by combining it with a method for identifying significant changes in the movement pattern similar to the likelihood-based behavioural change point analysis [90] (Fig. 3h.). The dBBMM, therefore, allows the Brownian motion variance parameter *σ*^*2*^_*m*_ to change across an animal’s path, which is fixed in the BBMM, improving UD estimation and allowing to infer behavioural changes along an animal’s track [119]. This process takes subsets of location points and evaluates for breakpoints or changes in the motion variance parameter. For this, a sliding window of w locations in size is placed on a subset of locations, and a margin size (*m*) of at least three locations is left on each end of the window, which is not used in the estimation. First, a single *σ*^*2*^_*m*_ value is calculated for the whole window, which is then split into all possible pairs of segments. Additional pairs of *σ*^*2*^_*m*_ values are then calculated for each split window. Then Bayesian information criterion values are calculated for each model and the case with the lowest value is selected. If the case of a single *σ*^*2*^_*m*_ is favoured, the value is assigned to the entire window. If a behavioural break is favoured, the calculated *σ*^*2*^_*m*_ values for each split window are assigned to the respective track segment on each side of the break [119]. Sliding this window across the track produces several *σ*^*2*^_*m*_ values for each segment, which are averaged to obtain one value per segment [119]. Then, UDs are calculated as in the BBMM [102], but more realistic estimations of space use are obtained because a flexible *σ*^*2*^_*m*_ is less biased, for example by not overestimating UDs during resting phases [119]. The selection of w and m is done by the researcher(s) and should follow biologically grounded criteria, although the authors provide some guidelines [119]. Increasing w improves the robustness of the variation parameter calculation, giving more stable estimates, albeit reducing sensitivity to short-term changes in *σ*^*2*^_*m*_. On the other hand, increasing m allows to detect weaker breakpoints, but the larger margins reduce the number of locations to detect breakpoints [119]. The variation of *σ*^*2*^_*m*_ can be used to infer the animal’s behaviour [119], for example, to identify circadian or seasonal patterns in habitat use from fine-scale movement variations [28, 119]. The dBBMM offers an automated process to analyse behavioural changes on a track without requiring external information to classify them (which can be laborious and at times not possible), making it a more objective and repeatable process [119]. However, the simplicity of using *σ*^*2*^_*m*_ to detect behavioural changes (turning angles, speed, step length) has the disadvantage that it can potentially assign similar *σ*^*2*^_*m*_ to very different behavioural states, which needs to be considered during the interpretation of results [119]. The dBBMM performed better than the BBMM at estimating home ranges with irregular sampling schemes and the variable UD allowed not to overestimate space use during resting phases or assign unrealistically high confidence intervals to migration segments [119]. It has also been shown to perform better than MCP and fixed KUD under low-resolution sampling [176].

##### Time-geographic density estimation

Time-geographic density estimation (TGDE) [50] combines time geography, the study of the movement of objects over time [92, 133], with statistical density estimation to generate a continuous probability density surface for a moving object over a fixed time interval [50]. TGDE uses three elements to analyse movement over space and time: control points, space–time paths, and space–time prisms. Control points are the observed locations with their corresponding timestamp, and space–time paths of a moving object are created by tracing a straight line between adjacent control points. Finally, space–time paths are used to construct space–time prisms or potential path areas (PPA), which encompass the total space that was accessible to the animal during the trajectory between the two control points. This is calculated using -and constrained by- the location in space of the two control points (as start and end locations for all simulated paths), the time elapsed between them, and the specified maximum velocity. A two-dimensional space–time prism is called a geo-ellipse, the equivalent of KUD’s kernel, but is centered on the path rather than on each location [50]. Similarly, densities can be estimated in a similar way to KUD, as geo-ellipses are constructed using a function that calculates the space-use probability distribution. This function can be uniform (all areas in the geo-ellipse weight equally) or of linear decay (intensity is weighted and decreases with distance from the control points and space–time path) [50], among other possibilities [55, 56]. A KDE bandwidth equivalent is obtained by setting the intensity of use to 0 beyond the boundaries of the geo-ellipse, where it is assumed impossible for the animal to have been. This is estimated using the maximum distance travelled in each geo-ellipse, calculated using the specified maximum velocity and time between the two control points, and, unlike KDE’s arbitrarily chosen bandwidth, can be confidently estimated if the animal’s properties are known [50]. If velocity is kept constant, a fixed-velocity TGDE is obtained, and if it is allowed to vary, it results in an adaptive-velocity TGDE [55]. The latter is better suited for home range analysis because it allows velocity to match the animal’s activity, i.e., when in rest vs. in active movement [55].

TGDE is mainly sensitive to the geo-ellipse function, temporal tracking intervals, sampling scheme, and defined maximum velocity [56]. The selection of the geo-ellipse function determines how the intensity of use will be distributed, however, its effect on the results is more limited [56]. Areas with more intense sampling have less uncertainty between locations, and therefore space use areas are drawn closer to the space–time path, whereas areas with more sparse sampling have broader density surfaces as an outcome of larger sampling gaps [50, 56]. Finally, the user-defined maximum velocity can have considerable effects [50, 56]. A higher maximum velocity will allow the PPA to reach farther from the space–time path, thus creating larger geo-ellipses and estimates of space-use [56] and also increasing bias [55]. Nevertheless, lower velocities are not always the best choice [56]. This value can be either theoretical or an observed maximum speed and its suitability can be evaluated by checking if all control points fall within the complete PPA or if only a small proportion falls outside the 95% isopleth [56]. The TGDE process results in a continuous probability density surface of the animal’s spatial position, from which core area and home range isopleths can be drawn [56]. TGDE works in analogous ways to KDE, however, TGDE is based on movement trajectories from autocorrelated locations and generally performs better in simulations [55]. Other advantages are the exclusion of inaccessible areas, considering uncertainty in spatial position during unsampled periods, objective smoothing based on the animal’s velocity, and correct incorporation of uneven sampling intervals.

#### Network analysis-based home range estimation

Network analysis (NA) is a part of graph theory applied in many disciplines to describe relationships between discrete objects [203]. Networks are formed using two basic units: nodes (the objects), and edges, which are links between pairs of nodes. Nodes are commonly used to represent two types of objects when using acoustic telemetry data. Nodes can represent receivers and edges the movement of tagged individuals between them, forming spatial networks, and nodes can also represent individuals and edges the interactions between them, which produces social networks [105, 106]. Network’s nodes and edges can be constructed with different complexity, and the most adequate representation will depend on several factors like the research question or the quality of the data. Simpler networks can be unweighted, in which only the presence or absence of a connection between nodes is shown by the presence or absence of an edge, or weighted, where an edge’s thickness is proportional to the frequency of connection between nodes. Edges can additionally be directed, which indicates the direction of interaction, i.e., whether a connection is inbound or outbound at a given node [107]. The structural importance of the elements in a network is described using centrality metrics. Some of the most common ones are node degree, or the number of edges connecting to a node; node strength, used to measure the sum of all connection weights at a node [12]; closeness, which uses shortest-path distances to describe how central a node’s position is in network space (the lower the sum of its edges or pathways connecting to other nodes, the higher the closeness) [195]; eigenvector, which indicates how well connected a node is to other well-connected nodes [20], and betweenness, which describes the contribution of a node to the connectivity between nodes in a network, or the number of times the node occurs in the shortest paths between all pairs of nodes [79]. Such metrics can be included in the network by adjusting the size of nodes and/or using a coloured scale, for example as depicted in [98, 105, 139]. If a spatial representation of the network is required, it can be obtained by plotting receiver positions on a map of the study site [70]. Networks can be constructed to answer specific questions, for example by grouping receivers by habitat or other environmental properties (e.g., depth) to study topics such as habitat use frequency, and movement patterns between areas of interest [63, 98]. Other types of tests like removal analyses can be made, where the importance of an area or individual and the effect of isolation are evaluated [63, 137]. On the other hand, networks that simultaneously depict two types of objects are called bipartite and, while not spatial in nature, are useful to represent associations between nodes of different classes [47]. In these representations, edges exclusively link nodes of different classes [47], for example connecting nodes that represent individuals to nodes representing receivers based on their visitation patterns [70].

Importantly, the network constructions hitherto mentioned are static because they aggregate data across time and assume edges to be permanent associations. However, the temporal dynamics of space use and sociality can be represented using dynamic networks, for which centrality metrics are also calculated [19, 107]. A type of dynamic network is the time-aggregated network, which uses a pre-defined time window that “slides” across the temporal axis of the data. This way, all movements encompassed by each window are collapsed into one network, creating a sequence of temporally ordered networks. Such constructions allow examining dynamic processes by assessing changes in network topography [19]. What type of network to use will depend on the data and research question [65].

Compared to probability distribution estimators such as KUD, one of the major differences with NA is that the latter uses a network of nodes to examine movement between receivers and does not require the calculation of UDs. Instead, metrics akin to KUD’s core areas (called core use receivers) and 95% isopleths ranges (general use receivers) are estimated. This can be advantageous when position estimates are not obtainable from the data, as results are similar to those of UD-based methods [121]. Network analysis also allows looking into movement within the array, identifying movement pathways across nodes, and highlighting paths that are more heavily transited or connect relevant areas [121]. The randomness in the network is also tested by developing specific null models, e.g. [44, 66, 126]. Like with other methods, NA and its associated centrality metrics also have limitations and can be subject to bias. For example, the design of the array needs to be accounted for in the analysis, as receiver positions in the array can influence their centrality, e.g., receivers on the edge of the array are less likely to be connected than those from the centre. Other considerations include that movement is three-dimensional and usually convoluted but is represented as a straight line in a network (such a simplification is also true for most space use estimators); a low number of nodes (low receiver coverage); receiver distribution; detection range of receivers and tags; and the temporal scale of movement is generally ignored [138]. Despite the applicability of NA to the study of space use using acoustic telemetry, not only in ecology but also in management and conservation [82, 105, 106], its use has been scarce compared to traditional home range estimators [121]. Network analysis can be conducted in the R environment with the R packages sna [27], igraph [45], and tnet [149]. Similarly, advances to facilitate the inclusion of time in NA have been proposed, by providing guidelines to analyse the temporal succession of network motif patterns [152] or by applying a moving window approach [21] using the bespoke R package netTS.^8^

As mentioned before, neighbouring receivers with overlapping detection ranges are often used to calculate positions/pseudo-positions for space use estimation. However, for spatial NA generally non-overlapping detection ranges are used, e.g., as in [105, 121]. Simultaneous (i.e., “duplicate”) detections at adjacent receivers are avoided with this configuration, as this can impact the centrality metrics. This presents a challenge when the goal is to both study space use and use NA using the same acoustic array. Two ways to work around this can be followed: either to modify the data to eliminate the overlap, or to create occupation pixels based on calculated positions/pseudo-positions before NA ([13, 152. To eliminate the overlap, the receivers connected by overlapping detection ranges can be combined into one node, e.g., as Stehfest et al. [185] did when creating receiver “curtains” to cover a passage. Alternatively, a subset of receivers can be selected, e.g., as done by Papastamatiou et al. [151], which requires careful consideration as it implies subjective data selection and could greatly alter the study design.

**Fig. 4:**
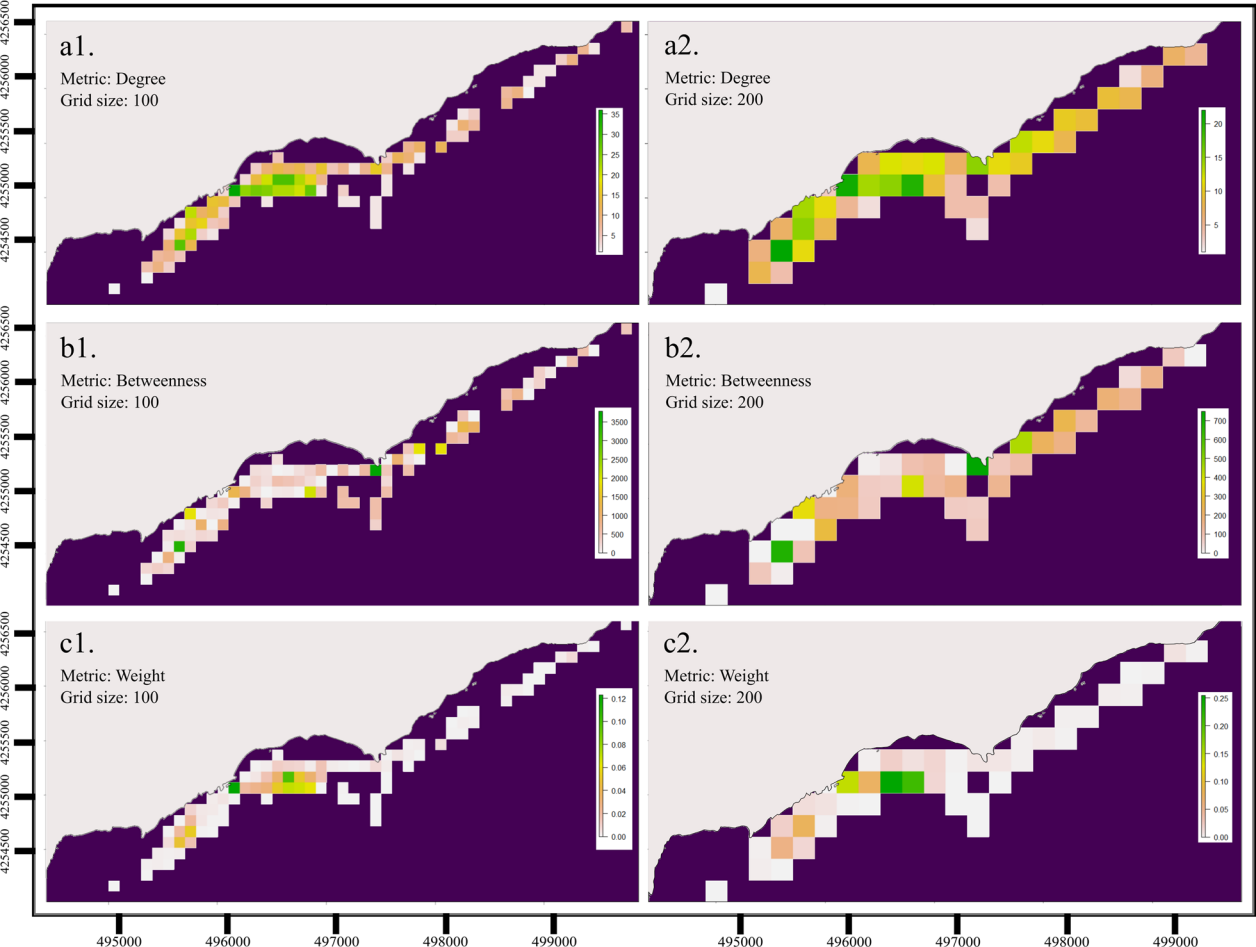
Examples of a spatial network created using a trajectory based on the COAs obtained using the *Dasyatis pastinaca* data set data. Shown are three centrality metrics (degree, betweenness, and weight) using two grid resolutions (100 and 200) highlighting the influence of grid resolution on network metrics. Degree: number of different pixels a pixel is connected to; Betweenness: number of shortest paths going through a pixel relative to the total number of shortest path (the importance of a pixel in the organization of flows in the network); weight: number of locations within a pixel. To investigate space use, degree can act as a measure of connectedness indicating spatial hubs where most movements depart from or/and arrive to; betweenness is a measure of connectivity and indicates bridge patterns in the network (i.e., corridors), and weight is a measure of residency or relocation density. The trajectory was created with adehabitatLT, the network metrics with moveNT. Image edited posteriorly for publication using Inkscape 1.1

**Fig. 5:**
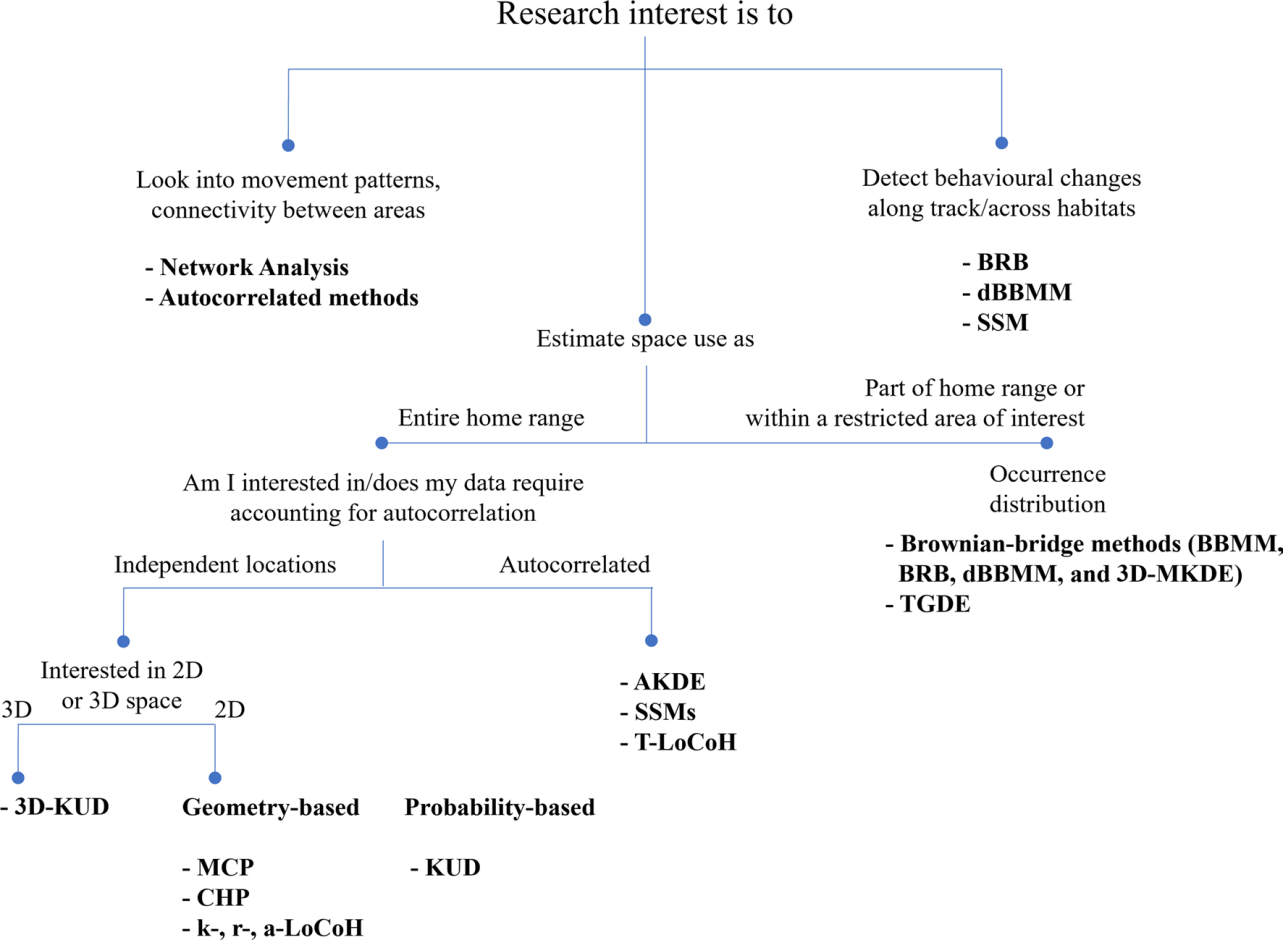
Decision tree with the described space use estimation methods, organized considering some of the questions they might be used to answer. BRB = Biased Random Bridges; dBBMM = dynamic Brownian Bridge Movement Model; SSM = State-Space Model; 3D-MKDE = Three-dimensional movement-based Kerned Density Estimation; TGDE = Time-Geography Density Estimation; 3D-KDE = Three-dimensional Kernel Utilization Distribution; MCP = Minimum Convex Polygon; k-, r-, a-LoCoH = Local Convex Hull methods; KUD = Kernel Utilization Distribution; AKDE = autocorrelated Kerned Density Estimation; T-LocoH = Time Local Convex Hulls

On the other hand, maintaining the overlapping receivers implies working with positions or pseudo-positions, and requires transforming raw movement data into a format that can be automatically analysed with network metrics. For this, the animal coordinates that make up the trajectory can be rasterized on a spatial grid, whose cells will become nodes and the movement between them the edges ([13, 152 (Fig. 4). Different grid resolutions affect the topological structure of the resulting network, which can be evaluated by using a range of grid resolutions to build and compare several networks to find the optimal resolution [152] (Fig. 5).

#### Three-dimensional KUD

Most movement ecology studies explore space use in two dimensions [120] and, when data on vertical movements (depth) are collected, they are generally analysed separately from horizontal movements. This precludes examining space use in the same number of dimensions aquatic animals usually move, which would be more complete and produce a more realistic understanding of their movements [181]. Three-dimensional position estimates operate by adding a third dimension to the calculation of pseudo-positions using COAs [181], or any method described above. For example, a basic 3D position using VPS is calculated in a process similar to the 2D equivalent, which locates the intersection of a set of hyperboloids (each one constructed by tracing a line with a constant range difference between pairs of receivers of known position) and a plane (defined by transmitter depth). The most common method of estimating space use volume is a three-dimensional version of KUD (3D-KUD) available in the R-package ks [58]. To build a 3D-KUD, first, a three-dimensional cube is created and gridded. This results in a cube in space that contains a user-defined number of three-dimensional pixels known as voxels. A density estimate is then calculated for each voxel, for which a bandwidth value is selected. Utilization distributions can then be visualized at different isopleths, like 50% 3D-KUD and 95% 3D-KUD. As in two-dimensional KUD estimation, under-or oversmoothing are consequences that can arise from inappropriate bandwidth selection [41]. Sample code to estimate 50% and 95% 3D-KUDs is available in the Appendix of [181] and [41]. Considering that marine species live and move in three dimensions, this approach can be a better fit which offers more ways to explore and analyse the data [122, 193]. 3D-KUD estimation can include three-dimensional topography [8]. A movement-based 3D space use estimator using Brownian bridges was also developed to estimate UDs [191], based on the raster [101] and Rcpp [59] R packages. Finer insights into space use of animals can be obtained compared to two-dimensional methods, like a fuller understanding of diel movement patterns [193], differences in home range size relating to intraspecific differences such as sex [122], the role environmental factors such as thermoclines can have as barriers [7], and the susceptibility of animals to certain types of fishing gear [181]. Additionally, estimating range overlap in 2D space can be prone to overestimation, as including a vertical dimension (depth) has shown that overlap can be reduced, like animals in the same location might be at different depths [38, 41, 181].

This approach, although a more realistic reflection of animal movement, is also more complex to implement and implies greater challenges. To date, 3D space use estimation has not been as widely used as its 2D counterpart for reasons such as a historical lack of suitable analytical tools [181]. Also, tags equipped with pressure sensors are required, information which is measured at the time of signal emission and sent along with the signal ID to estimate tag depth. Usually, receiver depth is also required. However, recently improved statistical analyses and capacity to improve movement data exploration render it a central part of future research [193].

## Discussion

Technological advances and the increased complexity and refinement of analytical methods have provided unprecedented insight into the study of animal movement in aquatic environments using acoustic telemetry [103]. However, quantifying a complex and continuous phenomenon is a challenging task. In the study of animal movement, a common objective is to estimate space use, for which several methods exist, some of which have been described here. These methods are commonly analysed using the R software [166], a free, open-source software in which research can be conducted in a reproducible way. It runs on all common operating systems and has a growing community constantly developing and updating bespoke code packages (Table 1). Further reading can be found in the review by Joo et al. [110] on R packages to analyse movement data, although not limited to estimation methods that use acoustic telemetry data.

**Table 1:**
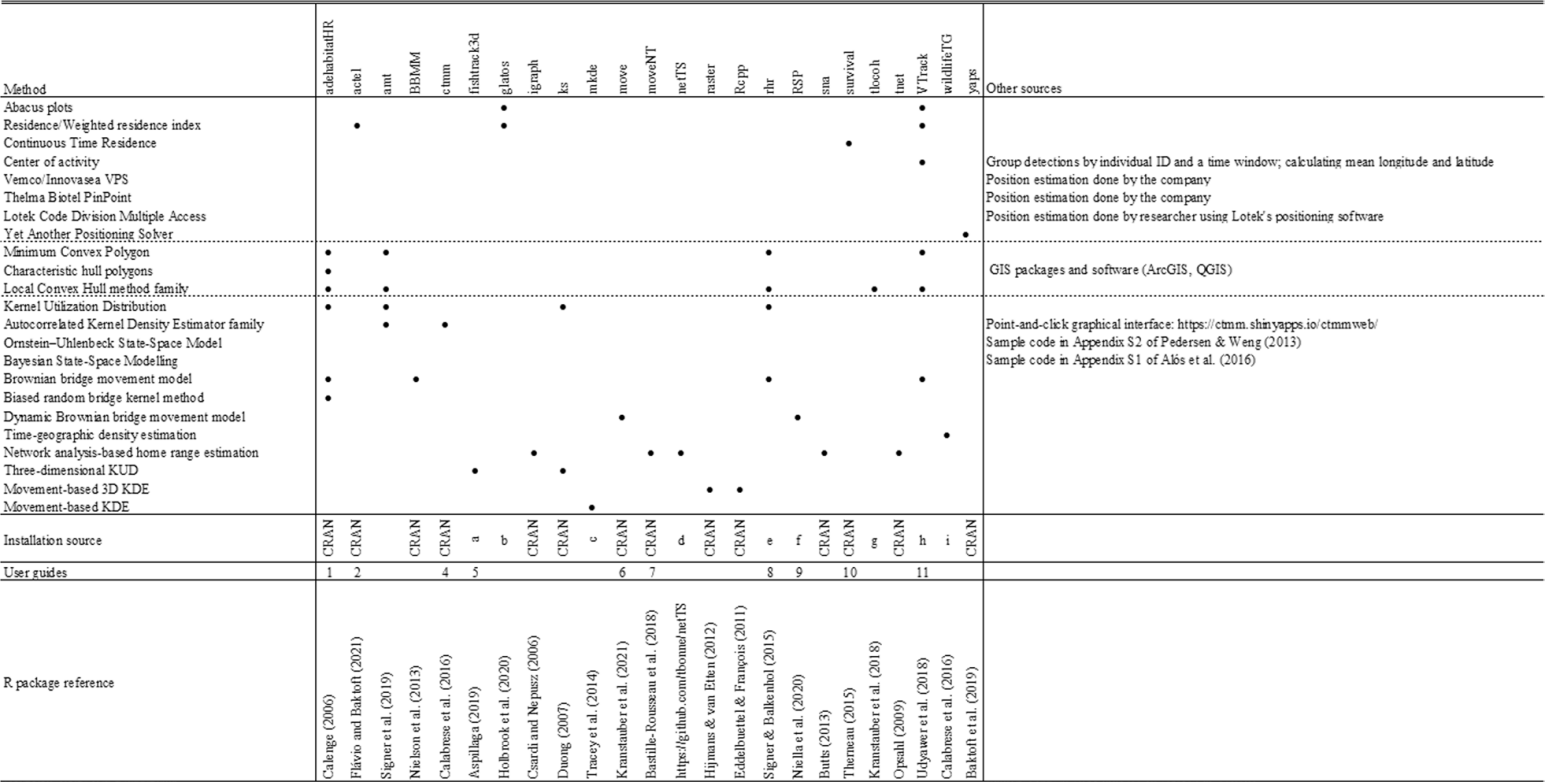
Table of available R packages that implement the described methods for positioning, residency, and space use estimation. Other software alternatives are also mentioned

a. GitHub (https://rdrr.io/github/aspillaga/fishtrack3d/)

b.GitLab (https://gitlab.oceantrack.org/GreatLakes/glatos/-/wikis/installation-instructions)

c.Removed from the CRAN repository and archived: https://cran.r-project.org/src/contrib/Archive/mkde/

d. GitHub (https://github.com/tbonne/netTS)

e. R-Forge (https://rdrr.io/rforge/rhr/)

f. GitHub (https://rdrr.io/github/YuriNiella/RSP/)

g. R-Forge (https://rdrr.io/rforge/tlocoh/)

h. GitHub (development version: https://github.com/RossDwyer/VTrack)

i. GitHub (https://github.com/jedalong/wildlifeTG)

1. https://cran.r-project.org/web/packages/adehabitatHR/vignettes/adehabitatHR.pdf

2. typing “browseVignettes(“actel”)” in R / https://CRAN.R-project.org/package=actel

3. Signer & Fieberg [175]

4. https://github.com/aspillaga/fishtrack3d/blob/master/vignettes/modelling_ud3d.Rmd

5. https://ecoisilva.github.io/AKDE_minireview/code/AKDE_R-tutorial.html

6. https://cran.r-project.org/web/packages/move/vignettes/move.html

7. http://www.spamwell.net/index.php

8. typing "browseVignettes("RSP")" in R/http://127.0.0.1:24834/session/Rvig.17a035e11a3a.html

9. Vignettes in https://CRAN.R-project.org/package=survival

10. https://vinayudyawer.github.io/ATT/docs/ATT_Vignette.html

Notwithstanding the advances in the field, there is no perfect, all-purpose space use estimator because there is no perfect way of measuring space use, and all methods have different properties and caveats [117, 163]. This contrasts with the general assumption that space use estimators have, which is considering the data to be composed of true locations, therefore becoming a source of uncertainty/error. Therefore, this decision process is not an easy task, as many factors must be considered. The suitability of each method depends on their particular characteristics and a combination of factors such as the research questions, properties of the data like gaps, resolution and monitoring time, study design and equipment, study species, and researcher experience [35, 69, 120], 142, 170, 175. The performance of estimators can be compared, for example, using the standardised workflow in the amt R package by Signer & Fieberg [175] that compares some of the most commonly used home range estimators (MCP, LoCoH, KDE and AKDE).

The most traditional estimators such as MCP and KUD are commonly favoured [120] over newer, likely better-suited ones for a variety of reasons [175]. The higher familiarity and more frequent use of the former might convey a greater sense of confidence and preclude from considering others. For most researchers, these reasons might at least presently exceed the analytical advantages offered by newer methods, which might appear too complex and of higher operational costs. For example, the wide application of SSMs in ecology had been impeded by computational limitations until some time ago [153], while three-dimensional methods, despite offering a more realistic estimation of the movement of animals [181], have their more extensive application hindered by the higher implementation costs and required analytical power. However, several of the newer, more complex methods offer guidelines and even R code to analyse data, for example for AKDEs [178], B-SSM [5], dBBMM^9^^,^ 10, and 3D-KUDs [41, 181].

One of the most important factors affecting an estimator’s efficiency is its compatibility with the question(s) driving the study and deciding on a method should be done with this in consideration. A table of key steps and considerations was provided by Fieberg & Börger, [69], as the properties of an estimation method can help highlight the aspects of the data that are of interest. For example, SSMs [153] and the Brownian bridge-based methods BRB and dBBMM are useful to explore behavioural changes in movement [176, 177]. Brownian bridge-based methods allow for studying changes in movement patterns and space use over different timescales (diel, seasons) or across different habitats (to forage, travel, rest) [90, 109, 136]. Identifying movement trajectories inside the activity space, which traditional kernel analyses do only superficially [121] is better done using methods that construct trajectories by including the time of locations in addition to their spatial distribution, like T-LoCoH and Brownian bridge-based methods. This approach enables the creation of paths and connecting areas that are important to the animal. Network analysis provides a different approach to this type of question, allowing for the exploration of movement patterns, detect important pathways and assess associations between individuals and habitat types inside the acoustic array, improving the understanding of space use and therefore a valuable analytical complement to probability density-based methods [47, 121]. Network analysis can provide metrics for each receiver that can be compared across time or among individuals (provided receivers have non-overlapping detection ranges). Network analysis also provides an alternative approximation to the estimation of core areas and home ranges while lacking area biases such as positive bias from boundary crossing and the need of calculating pseudo-positions [121]. Nevertheless, this method is not as commonly applied as others, greatly reducing across-studies comparisons [121].

Accounting for boundaries to movement might be important in some study areas, like when the receiver array is placed in rivers, fjords, or in proximity to coastlines, where large overlaps between space use estimates and land might occur, leading to the overestimation of space use. Limits to movement can also have biological origins such as territorial behaviours [15]. A common approach to remove densities estimated over unusable areas is to manually clip this portion of the area estimate after space use estimation, redistribute the density so it sums to 1 again and redraw the utilization boundaries. Alternatively, estimators that produce area estimates with less overlap can be favoured over others. Polygon-based methods like CHP and LoCoH-based methods present advantages over MCP and KUD because they accommodate boundaries and holes inside the estimated area [54, 83]. Similarly, among probability density estimators, movement-based methods generally deal better with barriers than the traditional KUD estimator because of the way density is modelled to fit the movement of the animal. Additionally, some R packages allow accounting for boundaries by explicitly including one during modelling. Examples of this are adehabitatHR [31], which allows creating a SpatialLines object to define an impenetrable barrier, RSP [143], which requires a shapefile with the topography of the study site to estimate the shortest path between consecutive detections, and in 3D-KUD methods [8].

Similarly, not all methods are equally suited to estimate home range. This will depend not only on the estimator but also on other factors such as the properties of the data and study design. Brownian bridge models are not true home range estimators but occurrence or trajectory estimators [75, 76]. They model probability distribution between successive pairs of locations and do not perform range prediction into the future, creating a trajectory with an associated uncertainty of where the animal was during the monitoring period [76]. This approach suits situations where the entire home range is most likely not being recorded, such as when the interest lies in characterizing space use during a restricted time frame and/or inside a limited area.

Furthermore, following Burt’s [26] definition of home range, it is important to ensure the extent of the animal’s home range is covered during the tracking period [146]. The time it takes for an individual to cover its home range can vary greatly [42] depending on the species, size, behaviour, or other characteristics, therefore it is crucial to adjust the monitoring period accordingly. Such range resident behaviour can be tested by constructing a variogram that seeks asymptotes in the extent of space use over time [29, 73, 173]. The time needed to reach asymptotic space use can be variable, for example depending on the species, e.g. [35].

If no asymptote is reached, home range estimation might not be the appropriate method to analyse the data set. This can occur for example if the animal is transiting through the study area instead of remaining, being only briefly within detection range or because the monitoring period is too short, or the acoustic array only partially covers the actual home range. In such scenarios, the use of occurrence estimators (i.e., to answer the question “where has the animal been during the tracking period?”) might better fit the data [76].

If the extent of the animal’s home range is effectively covered, whether the estimator operates under the assumption of IID or autocorrelation is important to consider [120, 146, 173]. Independent data points are obtained by sampling at intervals that are large enough to ensure they are not autocorrelated, which is habitually over timescales greater than the time an individual needs to cross its linear home range. The number of such locations in a data set is referred to as effective sample size. Larger effective sample sizes are preferred, so the study duration should ideally span for much longer than the home range crossing time [72, 146]. This differs from (and is sometimes confounded with) absolute sample size, which is the total number of locations in a data set regardless of the sampling frequency [72, 146]. The time of statistical independence (time interval after which two subsequent locations are statistically independent) [187] can be tested, for example, with the R package rhr [173].

However, autocorrelation is an intrinsic property of animal movement [72, 146] and most studies do not include it [120]. Moreover, the assumption of IID is incompatible with (most) movement data sets, because autocorrelation intensifies with an increasing sampling frequency [48, 187], which is especially relevant when working with acoustic telemetry data [103]. Modern acoustic telemetry techniques allow the collection of data at very high frequencies, so the number of locations corresponds to the absolute sample size [72, 146]. Traditional home range estimators usually assume independent data points, like most polygon-based methods and KDE, hence movement data obtained with acoustic telemetry is incongruous with this underlying assumption.

A way to comply with this assumption is to coarsen the sampling period to space out locations in time to be considered independent [76]. However, removing data to fulfil this assumption results in tremendous amounts of data loss, and subsampling tracking data is generally not recommended [173]. Alternatively, most modern estimators account for autocorrelation by fitting an underlying movement model to the data, making them more suitable to work with acoustic telemetry [29, 146]. Of such estimators are the recently developed series of AKDEs [72, 76, 77] and state-space models [5, 156]. The former methods correct biases that are frequently encountered when working with autocorrelated movement data, like failing to consider autocorrelation, small effective sample sizes, and missing or irregularly sampled data, producing comparatively unbiased home range estimates relative to traditional methods [146]. Since restrictions to movement are not explicitly included in the estimation process, AKDE can still present drawbacks such as positive bias from spilling over boundaries, but corrections can be made to address this [146, 178]. AKDEs have also been improved in user-friendliness, as they were compiled into one publication with guidelines and an R script to facilitate their use [178]. Furthermore, in addition to the R package ctmm [29], a point-and-click graphical interface is available and can be installed using the R console or used directly through a website [30].

With the advancement of tracking technologies and improved computational capacities, it is becoming easier for researchers to collect movement information in larger quantities and greater detail. The combination of these conditions presents the chance to take fuller advantage of the data by performing more complex analyses. Hull-based estimators discard much of the information, so to this end they should be avoided, and newer, more refined space use estimators should be preferred and set as the standard in the field.

### Active telemetry

Active acoustic telemetry, following the movements of tagged individuals from a moving vessel using a hydrophone, is a methodology that has been applied to track animals for a long time, e.g., [34]. Despite the finer resolution of active tracking [99], its use to inform the design of a passive acoustic array [68], and recent technological advances like replacing humans with autonomous underwater vehicles to do the tracking [204], passive acoustic telemetry remains the preferred method today. In active tracking, data collection is usually done on one focal individual at a time and its duration directly depends on the researchers’ capacities and weather conditions. Because of this, tracking periods usually span from a few hours to a few days (not sampled continuously). Moreover, the presence of the monitoring vessel could disturb the tracked individuals and alter their behaviour [99, 114]. Nevertheless, most of the methods described here are applicable to active telemetry data sets, including minimum convex polygons [202], local convex hulls [80], kernel utilization distributions [68, 202], and the Brownian bridge movement model [131, 204].

## Summary

Technological advances in acoustic telemetry and the increased complexity and refinement of analytical methods have provided unprecedented insight into movement in aquatic environments [103]. The capability of collecting animal movement data has also increased from tens of locations by direct observation or radio collars, to thousands of high-frequency fixes from long-term monitoring using acoustic arrays [103, 113]. However, quantifying a complex and continuous phenomenon such as animal movement is a challenging task. Correct space use analysis requires careful selection of the estimation method, as alternatives have diversified over the years, and each has different properties and presents different advantages and drawbacks. This decision is influenced by factors like the type of data, study design, researcher experience, monetary restrictions and processing capacities, and the questions behind the research. The present work and the additional file showcase some of the estimators and methodologies used to evaluate their performance and execute analyses (Additional file 1).

## Supplementary Information

**Additional file 1.** Description of three space use estimation methods not included in the main text: Product Kernel Algorithm, Network-based Kernel Density Estimator, and the Lattice-based density estimator.

## Data Availability

Available upon request.

## Abbreviations

AKDE: Autocorrelated kernel density estimation
AKDE_C_: Area-corrected AKDE
BBMM: Browninan bridge movement model
BRB: Biased random bridge kernel method
B-SSM: Bayesian State-space model
CAT: Continuous absence time
CDMA: Code division multiple access
CHP: Characteristic hull polygons
COA: Center of activity
CTR: Continuous time residence
D: Diffusion coefficient
D_d_: Total number of days the animal was detected
D_i_: Number of days between first and last detection
D_t_: Time between tagging date and end of last monitoring day
dBBMM: Dynamic Brownian bridge movement model
DH: Habitat-specific diffusion coefficient
DOP: Dilution of precision
h: Smoothing factor, bandwidth
HDOP: Horizontal dilution of precision
HPE: Horizontal Position Error
IID: Independent and identically distributed
I_R_: Residency index
I_WR_: Weighted residency index
KUD: Kernel utilization distribution
LoCoH k-, r-, a: Local convex hull, nearest-neighbour-, radius-, adaptive-
m: Margin size
MBP: Maximum blanking period
MCP: Minimum Convex Polygons
MKDE: Movement-based kernel density estimation
MSHC: Minimum spurious hole covering
NA: Network analysis
NNCH: Nearest neighbour convex hull
OU: Ornstein–Uhlenbeck
OUF: Ornstein–Uhlenbeck Foraging
OU-SSM: Ornstein–Uhlenbeck State-space model
PDF: Probability density function
PPA: Potential path areas
RI: Reliability index
s: Scaling parameter
SPP: Spatial point process
SSM: State-space model
TDOA: Time difference-of-arrival
TGDE: Time-geographic density estimation
T-LoCoH: Time Local Convex Hull
T_max_: Upper recording time limit
TOA: Time of arrival
TSD: Time-scaled distance
UD: Utilization distribution
VPS: Vemco positioning system
VUE: Vemco user environment
w: Sliding window
wAKDE_C_: Optimally weighted and area-corrected AKDE
YAPS: Yet another positioning solver
σ^2^_m_: Brownian motion variance parameter

## Footnotes

1 https://support.vemco.com/s/downloads.

2 http://www.thelmabiotel.com/service/pinpoint/.

3 http://www.thelmabiotel.com/software/.

4 http://github.com.

5 https://www.arcgis.com/index.html.

6 https://www.qgis.org/en/site/.

7 https://ecoisilva.github.io/AKDE_minireview/code/AKDE_R-tutorial.html.

8 netTS: https://github.com/tbonne/netTS.

9 R-package RSP vignettes: http://127.0.0.1:12148/session/Rvig.512c13945f85.html for which data is prepared using actel [71]: https://CRAN.R-project.org/package=actel.

10 R-package move vignettes: https://cran.r-project.org/web/packages/move/vignettes/browseMovebank.html.
