# Supplementary material for: Residency and space use estimation methods based on passive acoustic telemetry data
Source: Mov Ecol. 2023 Mar 1;11:12. doi: 10.1186/s40462-022-00364-z (PMC9976422; doi:10.1186/s40462-022-00364-z)
Supplement: Supplementary file 1 — Additional file 1. Description of three space use estimation methods not included in the main text: Product Kernel Algorithm, Network-based Kernel Density Estimator, and the Lattice-based density estimator. [file 40462_2022_364_MOESM1_ESM.docx]

# Supplementary file

# **Density distribution probabilistic home range estimators**

## Product Kernel Algorithm (PKA)

The PKA extends van Winkle’s (1975) original UD that defines the frequency distribution of an animal’s occurrence in two-dimensional space to also include elevation as the third dimension of space, and time (Keating and Cherry, 2009). Time can be handled in different ways depending on the questions being asked. For example, to evaluate temporal changes over years, time can be treated as linear. If repeatable patterns are to be investigated, like seasonal migrations or daily activity patterns, time can be given circular nature. For this, time is recorded as the day of the year and has an interval of [-π, π] radians (Keating and Cherry, 2009). This type of UD analysis uses a kernel based on the wrapped Cauchy distribution (Batschelet, 1981) that seeks to appropriately weight moments in circular time that are proximate to each other. As an example, when estimating the density of occurrence on the 365^th^ day of the year, the observations from the 1^st^ day of the year should have a higher weight than observations from five days before (360^th^ day) (Keating and Cherry, 2009).

## Network-based Kernel Density Estimator (NKDE)

First proposed by Downs and Horner, (2007), the NKDE uses a network and non-Euclidean distances (i.e., not in a straight line) to estimate space use. This seeks to improve the assumption of Euclidean distances in KDE. NKDE differs from the Kernel Density Estimator (KDE) in that location points are viewed as a sample taken from an animal’s network of movement trajectories, which are in turn used to recreate this underlying movement network on which KDE estimation is subsequently based. The network is constructed using Delaunay triangulation, which connects locations by creating triangles with the smallest angles possible. This way, long-distance Euclidean trajectories are avoided, as straight paths are only generated between location points that are close in space. Paths between distant locations are formed by intermediate locations rather than directly connecting them with a straight line (Downs and Horner, 2007b). NKDE adapts KDE to calculate space use using the constructed network as a base instead, i.e., distance-weighting is a function of the network and not Euclidean space. This is argued to better fit the underlying point distribution and more realistically identify areas of high intensity, although based on preliminary results. This could present advantages in cases of uneven space use, in the presence of barriers or corridors, or in an individual’s preference of transiting through certain areas (Downs and Horner, 2007b).

## Lattice-based density estimator (LDE)

The LDE (Barry and McIntyre, 2011) seeks to improve space use estimation by including boundaries to movement and holes within space use areas (i.e. unused areas), which are shortcomings of other traditional methods like KDE and Minimum Convex Polygon, e.g., (Getz and Wilmers, 2004; Powell, 2000). The LDE method first creates a polygon in the shape of the study area and fills it with nodes. The distance between nodes is user defined and should be as small as possible, also defining the number of nodes in the grid. (Barry and McIntyre, 2011). Nodes are then linked to their immediate neighbours in eight possible linking directions to form a lattice, which represents the possible movement directions from each node. The grid is then shaped to fit the study area by removing nodes and/or links in places with boundaries to movement or geographic features, like shorelines, islands, or strips of impenetrable land over which two nodes are linked, or following another type of resoning the researcher might have. This way, areas outside of the polygon that conforms the study area, which have neither nodes nor links, are assigned a density of zero and are not considered in space use estimation (Barry and McIntyre, 2011). A node can therefore have between one to eight neighbouring nodes, depending on its place in the grid. Then a Brownian motion-based random walk restricted to the previously defined boundaries is set on the lattice. However, unlike most Brownian-bridge based models, LDE modeling assumes independent relocations, and the path between two relocations is not constrained at the start and end. Random walks begin at each node where an observed location point is placed, and at the beginning (at *k*=0 steps) the probability density will be distributed only in those nodes. Then these disperse to other nodes with each step of the random walk. For each step’s direction, the random walk considers the number of neighbours surrounding a point (the possible directions of movement) and parameter *M.* This parameter ranges from 0 to 1 and indicates the probability that the random walk will remain at the current location. The lower the value, the more steps are needed to achieve the same degree of smoothing (Barry and McIntyre, 2011). This is used to generate a transition matrix with the probabilities of a step’s action, to remain at the present location or move to one of the neighbouring locations. The fewer neighbours a given node has, the higher the probability to remain at the same location will be, and therefore the node will be of higher density. However, the configuration of the transition matrix can be changed, for instance, to a uniform probability of remaining at the node across the study area, to vary flow rates in different sectors of the study area, or to promote movement in certain directions (Barry and McIntyre, 2011). Density estimations result from the sum of all random walks that originated from the observed relocations. Smoothing of the density map is greater with an increasing number of steps, therefore selecting *k* is akin to defining bandwidth. By seeking the value which minimizes the unbiased cross-validation criterion (Sain et al., 1994) the optimal number of steps value can be obtained (Barry and McIntyre, 2011). LDE has been used to estimate the activity space of sharks within bays of complex topography (Kneebone et al., 2012; Legare et al., 2015). An R-package called *latticeDensity* was developed to use this space use estimator^[[1]](#footnote-1)^ (Barry and McIntyre, 2011).

## References

Batschelet, E.M., 1981. Circular statistics in biology. Academic Press, New York, New York, USA.

Downs, J.A., Horner, M.W., 2007a. Characterising Linear Point Patterns, in: Proceedings of the GIScience Research UK Conference. Maynooth, Ireland.

Downs, J.A., Horner, M.W., 2007b. Network-based Home Range Analysis Using Delaunay Triangulation, in: 4th International Symposium on Voronoi Diagrams in Science and Engineering (ISVD 2007). IEEE, Glamorgan, UK, pp. 255–259. https://doi.org/10.1109/ISVD.2007.31

Getz, W.M., Wilmers, C., 2004. A local nearest‐neighbor convex‐hull construction of home ranges and utilization distributions. Ecography 27, 489–505. https://doi.org/10.1111/j.0906-7590.2004.03835.x

Keating, K.A., Cherry, S., 2009. Modeling utilization distributions in space and time. Ecology 90, 1971–1980. https://doi.org/10.1890/08-1131.1

Kneebone, J., Chisholm, J., Skomal, G., 2012. Seasonal residency, habitat use, and site fidelity of juvenile sand tiger sharks *Carcharias taurus* in a Massachusetts estuary. Marine Ecology Progress Series 471, 165–181. https://doi.org/10.3354/meps09989

Legare, B., Kneebone, J., DeAngelis, B., Skomal, G., 2015. The spatiotemporal dynamics of habitat use by blacktip (*Carcharhinus limbatus*) and lemon (*Negaprion brevirostris*) sharks in nurseries of St. John, United States Virgin Islands. Mar Biol 162, 699–716. https://doi.org/10.1007/s00227-015-2616-x

Powell, R.A., 2000. Animal home ranges and territories and home range estimators, in: Boitani, L., Fuller, T.K. (Eds.), Research Technologies in Animal Ecology - Controversies and Consequences. Columbia Univeristy Press, New York, pp. 65–110.

Sain, S.R., Baggerly, K.A., Scott, D.W., 1994. Cross-Validation of Multivariate Densities. Journal of the American Statistical Association 89, 807–817. https://doi.org/10.1080/01621459.1994.10476814

1. [https://CRAN.R-project.org/package=latticeDensity](https://cran.r-project.org/package=latticeDensity) [↑](#footnote-ref-1)
